# Multiscale investigation of CO_2_ capture in 1,2,4-triazolium-based ionic liquids and porous copolymers using density functional theory and molecular dynamics

**DOI:** 10.1039/d6cp02655a

**Published:** 2026-09-04

**Authors:** Sebastian Anila, Jiayin Yuan, Alexander P. Lyubartsev

**Affiliations:** a Department of Chemistry, Stockholm University S-10691 Stockholm Sweden anilasebastian25@gmail.com alexander.lyubartsev@su.se

## Abstract

Capturing carbon dioxide (CO_2_) directly from the atmosphere has become an essential approach in efforts to address climate change, particularly within broader carbon capture and storage frameworks. Among the materials explored for direct air capture, ionic liquids (ILs) stand out as their chemical structure can be finely adjusted to optimise how they interact with CO_2_, including both capture and subsequent transformation. Building on this concept, poly(ionic liquid)s (PILs) have been developed as an advanced class of materials that provide greater structural stability and enhanced interaction sites, leading to more efficient CO_2_ uptake. This study investigates the CO_2_ capture performance of six 1,2,4-triazolium-based ILs and their corresponding porous copolymers, using a combined density functional theory (DFT) and all-atom molecular dynamics (MD) simulation approach. The DFT calculations show that CO_2_ adsorption is mainly governed by noncovalent interactions with the anions, while the functional groups attached to the 1,2,4-triazolium cations strongly influence the adsorption strength by increasing the availability of electron-rich interaction sites, as consistently evidenced by molecular electrostatic potential (MESP) analysis. The frontier molecular orbital (FMO) analysis and quantum theory of Atoms-in-molecules (QTAIM) analysis confirms that these functional groups do not merely contribute sterically but actively modulate the electronic distribution responsible for stabilizing the adsorbed CO_2_ molecules. MD simulations complement these results by capturing bulk thermodynamic and transport properties under realistic conditions. This integrated multiscale framework provides a comprehensive understanding of 1,2,4-triazolium-based sorbents and guides the rational design of next generation materials for efficient, regenerable, and scalable CO_2_ capture applications.

## Abstract 2

DFT and MD simulations reveal the molecular descriptors governing CO_2_ capture in functionalized 1,2,4-triazolium ionic liquids and porous copolymers, providing insights for the rational design of efficient CO_2_ sorbents.

## Graphical abstract

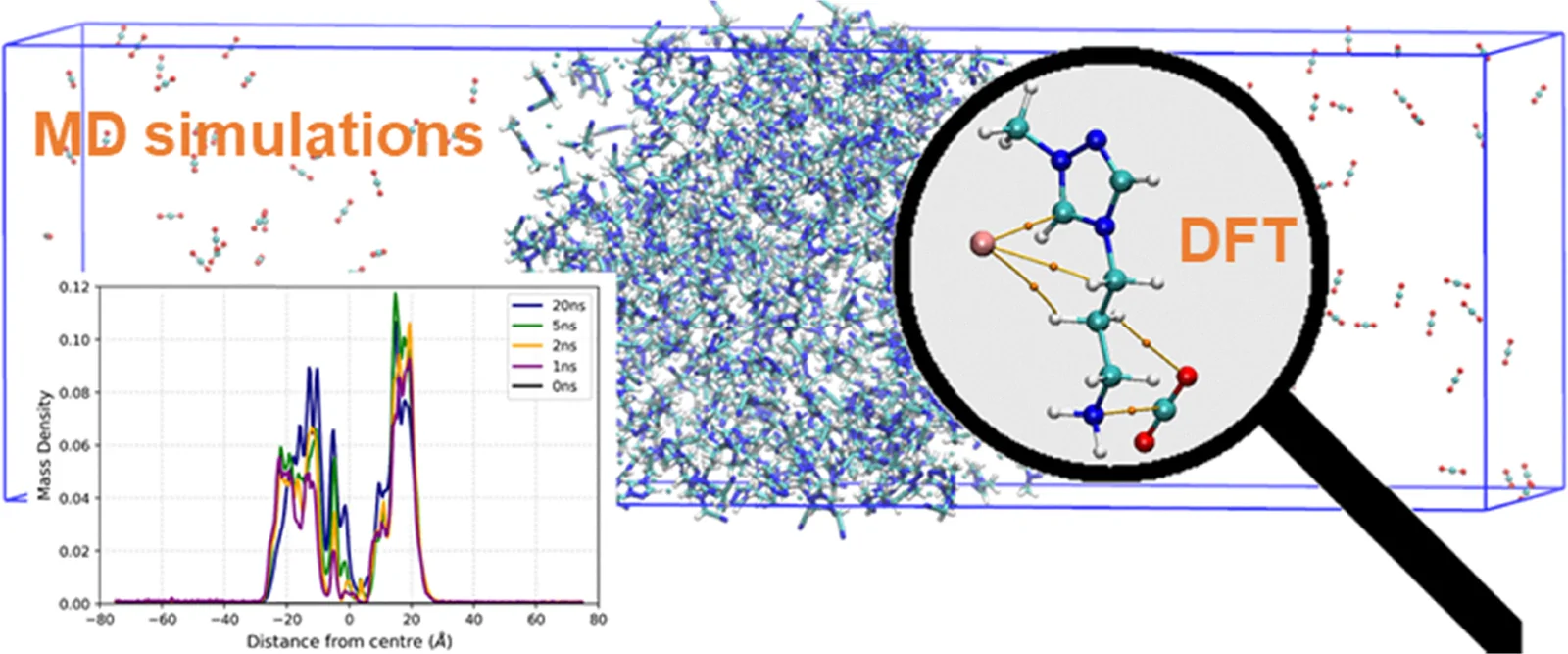

## Introduction

The accelerating and sustained rise of atmospheric carbon dioxide (CO_2_), the dominant anthropogenic greenhouse gas largely coming from fossil fuel combustion and industrial activity, is a major driver of global warming and climate change.^1–4^ According to recent reports, atmospheric CO_2_ levels have surpassed 420 ppm, compared to the pre-industrial levels of ∼280 ppm emphasizing the urgency for effective mitigation strategies.^5^ In this context, carbon capture, utilization, and storage (CCUS) technologies have attracted significant attention as practical approaches to reduce CO_2_ emissions while enabling its transformation into value-added chemicals and fuels.^6–8^ Conventional aqueous amine-based CO_2_ capture methods, though efficient, face issues like high regeneration energy, solvent degradation, corrosion, and volatility. These challenges have motivated the search for alternative materials with high CO_2_ affinity, stability, and variable properties.^9^ Among these ionic liquids (ILs), salts with melting points below 100 °C, have emerged as promising candidates due to their negligible vapour pressure, high thermal stability, wide electrochemical window, and structural tunability.^10–14^

Since the early demonstration of CO_2_ solubility in imidazolium-based ILs, extensive experimental and computational research has been conducted to elucidate their interactions with CO_2_ and optimise their capture performance.^15–18^ The ability to independently tune cations and anions gives ILs exceptional versatility, allowing their properties to be tailored for specific applications. In CO_2_ capture, the anion typically plays a dominant role through physical interactions or chemical binding, while the cation influences free volume, viscosity, and overall thermodynamics.^19,20^ Functionalized ILs, including those containing amine, hydroxyl, or heterocyclic moieties, have been shown to significantly enhance CO_2_ uptake and selectivity.^21,22^ Density functional theory (DFT) calculations have been extensively employed to quantify interaction energies, identify preferred binding sites, and analyse charge transfer between CO_2_ and IL components.^23–25^ In parallel, molecular dynamics (MD) simulations provide valuable insights into the dynamic behaviour of CO_2_ in IL environments, linking molecular-scale interactions to macroscopic properties.^26–29^

Among various IL families, nitrogen-rich heterocyclic cations have attracted growing interest. While imidazolium-based ILs are the most studied, 1,2,4-triazolium ILs have emerged as an important alternative owing to the presence of an additional nitrogen atom in the aromatic ring, which offers unique electronic and structural features.^30–35^ This structural modification alters charge distribution, hydrogen-bonding capability, and frontier molecular orbital (FMO) energies, influencing CO_2_ interactions, which suggest that triazolium cations offer a promising platform for designing advanced CO_2_-responsive materials. Despite the growing body of work on 1,2,4-triazolium ILs, systematic computational studies that correlate electronic properties, interaction mechanisms, and macroscopic capture performance are still relatively limited.

Poly(ionic liquid)s (PILs) combine favourable physicochemical properties of ILs with the mechanical robustness, ease of processing, and structural versatility of polymeric materials.^36–38^ In contrast to conventional porous frameworks such as metal–organic frameworks and covalent organic frameworks, whose CO_2_ affinity, stability, and regeneration behaviour depend strongly on their framework chemistry, PILs offer molecularly tunable CO_2_ interactions through their ionic components.^39,40^ By incorporating ionic moieties into polymer backbones or side chains, PILs offer enhanced stability, tunable porosity, and the potential for heterogeneous operation. Their high surface area, porosity, and improved gas diffusivity make them attractive for CO_2_ capture and conversion under milder conditions. Compared with monomeric ILs, PILs offer greater material strength, opportunities for heterogeneous catalysis, and variable microenvironments around ionic sites that influence CO_2_ interactions. Studies have demonstrated that PILs with porous architectures can achieve competitive or superior CO_2_ uptake, compared to their monomeric IL counterparts.^41–43^ Computational studies remain limited due to structural complexity, but recent computational investigations indicate that polymer architecture, ionic group distribution, and free volume significantly affect CO_2_ transport and binding.^44–47^

Incorporating 1,2,4-triazolium motifs into polymeric frameworks has recently attracted increasing interest as a strategy to combine the favourable CO_2_ interaction characteristics of triazolium cations with the structural advantages of porous polymers. Experimental studies have shown that triazolium-based PILs and porous polymer networks can exhibit enhanced CO_2_ affinity and promising catalytic performance in CO_2_ conversion reactions.^33,48^ Despite recent advances, a comprehensive molecular-level understanding of CO_2_ adsorption and transport within these materials remains insufficient. In particular, the effects of cross-linked porous architectures, ion-pair organization, and local polymer environments on CO_2_ binding and diffusion have not been systematically investigated. Thus, detailed computational studies integrating electronic-structure analysis with molecular-scale simulations are needed to establish structure–property relationships that can guide the rational design of competent triazolium-based CO_2_ capture materials.

In light of the above considerations, the present work aims to provide a comprehensive computational investigation using DFT and all-atom MD simulations to elucidate the CO_2_ capture performance in 1,2,4-triazolium-based ILs/PILs and their porous copolymers formed with divinylbenzene (DVB) as the cross-linkers. Here we explore how CO_2_ binds, moves, and behaves within these cross-linked porous copolymer networks at the molecular level and relate the obtained insights to existing experimental observations. Electronic-structure calculations are performed to determine energetically favourable CO_2_ adsorption configurations and to characterize the dominant interaction sites within the ionic species. To gain deeper insight into the nature of these interactions, molecular electrostatic potential (MESP), quantum theory of Atoms-in-molecules (QTAIM), and FMO analyses are performed. Complementarily, MD simulations are conducted to investigate the adsorption of CO_2_ from the gas phase into the copolymer networks and to examine the spatial distribution and organization of the various components within the system. The resulting molecular insights are expected to establish structure–property relationships that support the future development of advanced 1,2,4-triazolium-based materials for efficient CO_2_ capture and sustainable carbon management.

## Computational methods

### Electronic-structure calculations

Throughout this study, the ion pairs employed in the DFT calculations are treated as representative models of the monomeric IL/PIL units as well as the ionic moieties embedded within the corresponding porous copolymer networks. For convenience, these representative structures are collectively denoted as ILs. All the geometry optimisations were performed using DFT at the M06-2X/6-311++G** level with the Gaussian 16 suite of programs.^49^ The M06-2X functional is widely regarded as one of the most reliable methods for modelling intermolecular non-covalent interactions.^50,51^ Vibrational frequency analyses were subsequently performed to confirm the optimised geometries as true energy minima.

The interaction energies of the complexes were calculated using the super-molecule approach. For any two interacting subsystems A and B, the stabilization energy or the energy of interaction (Δ*E*) of the super-molecule C is defined as,

1 Δ*E* = *E*_C_ − *E*_A_ − *E*_B_

In this study, two forms of CO_2_ interaction energy were considered, defined as,

2 Δ*E*_1_ = *E*_Total_ − *E*_cation_ − *E*_anion_ − *E*_CO_2__

3 Δ*E*_2_ = *E*_Total_ − *E*_IL_ − *E*_CO_2__

where *E*_Total_ is the total energy of the IL⋯CO_2_ complex; *E*_IL_, *E*_cation_, *E*_anion_, and *E*_CO_2__ represent the energies of the ion-pair in the IL, cation, anion, and CO_2_ molecule respectively. Zero-point vibrational energy (ZPE) corrections were included to obtain more accurate results. Further, the basis set superposition error (BSSE) was corrected using the counterpoise approach of Boys and Bernardi.^52–56^ A negative Δ*E* indicates an exothermic process, with increasingly negative values reflecting stronger and more stable interaction configurations.

MESP analysis was performed at the same level of theory on optimised ion-pair structures to examine the electron density distribution and identify the most favourable sites for CO_2_ interaction. MESP is a real physical property which is directly related to the electron density function *ρ*(*r*).^57,58^ MESP at any point *r* in space, *V*(*r*), is defined by the equation,4
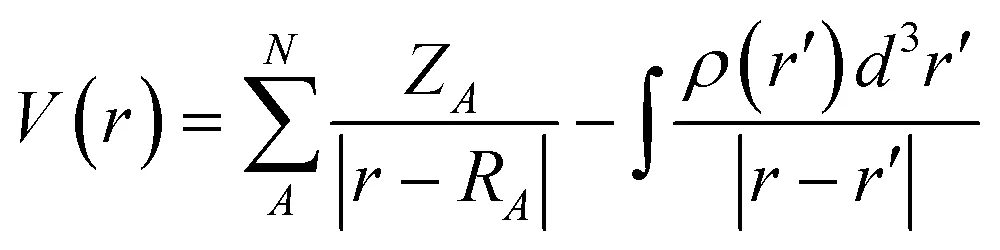
where *Z*_*A*_ is the charge on the nucleus located at a distance *R*_*A*_, and *ρ*(*r*′) is the electron density.^59^

Bader's QTAIM analysis, based on the electron density *ρ*(*r*) distribution, was employed to gain a deeper insight into the covalent and non-covalent interactions present in the complexes as well as interaction cooperativity.^60,61^ QTAIM plots contain bond paths, which are lines connecting the bonded nuclei through the distributed locally maximal electron density and the corresponding bond critical points (bcps).^62,63^ Atom-atom interactions are distinguished as covalent and non-covalent interactions based on the electron densities at the respective bcps (*ρ*_b_) as well as from the sign of the corresponding Laplacian of electron density (∇^2^*ρ*). Additional descriptors, including the sum of electron density at the bcps (Σ*ρ*_b_), the sign of the total Laplacian of electron density (Σ∇^2^*ρ*), and the sum of energy density (ΣH*ρ*) are also analysed for all the complexes. QTAIM properties were computed using the Multiwfn 3.8 program.^64^ The resulting output files processed with Visual Molecular Dynamics (VMD, version 1.9.3) to visualize bond paths and identify bcps within the QTAIM framework.^65^

### Molecular dynamics simulations

The polymeric model used in this work was constructed by assembling an oligomer composed of eight DVB units and two functionalized 1,2,4-triazolium groups. This oligomer geometry was optimised at the B3LYP/6-31G(d) level using Gaussian 16 and subsequently adopted as the structural unit representing the porous copolymer framework in the MD simulations.

Atomistic MD calculations were carried out using GROMACS 2020.1.^66^ Interactions between atoms were described through the GAFF force field, where Lennard-Jones parameters were directly adopted from the standard parameter set.^67^ Partial charges were obtained from HF/6-31G(d)//b3lyp/6-31G(d) electrostatic potential calculations and fitted using the restrained electrostatic potential (RESP) method as implemented in the Ambertools20 package.^68^ Non-bonded interactions were truncated at 16 Å,^44,69^ while long-range electrostatics were treated with the Particle Mesh Ewald scheme.^70,71^ Hydrogen-containing bonds were constrained using the Linear Constraint Solver (LINCS) algorithm.^72^ Periodic boundary conditions (PBC) were applied in all three directions. Simulation trajectories were visualized and analysed using VMD.^65^

The overall molecular dynamics simulation strategy adopted in this work is based on the methodology developed by Aghaie *et al.*^45^ and subsequently applied by Zhang *et al.*^44^ to investigate CO_2_ absorption in imidazolium-based ionic liquids. In the present study, this framework is adapted to porous cross-linked copolymers containing functionalized 1,2,4-triazolium ionic moieties. Compared with the previously studied ionic liquid systems, the polymeric architecture introduces heterogeneous ionic environments, restricted segmental mobility, and confined free volume, requiring modifications to the equilibration procedure while enabling a more detailed investigation of CO_2_ adsorption and transport.

#### Construction of the equilibrated copolymer model

The copolymer system, containing 40 oligomer chains and charge-compensating counter anions (Br^−^ or CH_3_COO^−^), was constructed by inserting multiple copies of the optimised oligomer (SI, Fig. S1) into a simulation box using the gmx insert-molecules utility in the GROMACS package. The initial configuration was then equilibrated to obtain a stable amorphous bulk phase. The equilibrated copolymer matrix, with dimensions of 4.8 × 4.8 × 4.8 nm^3^, served as the reference configuration for all subsequent CO_2_ adsorption simulations (Fig. S2(a)). Because relaxation in cross-linked polymer networks is considerably slower than in molecular ionic liquids, an extended annealing procedure was adopted. All the copolymer systems were relaxed in the NVT ensemble for 50 ns, with the temperature starting at 300 K, gradually increasing to 600 K within the first 5 ns, followed by gradual decrease to 300 K by 10 ns, and then maintained at 300 K for the remaining 40 ns. Further equilibration was conducted in the NPT ensemble for additional 50 ns with the system pressure kept at 1 bar. The extended annealing protocol improves conformational sampling and facilitates relaxation of the cross-linked polymer network prior to CO_2_ adsorption simulations.

#### Interfacial CO_2_ adsorption simulations

Following the above equilibration, the simulation box was expanded sixteen-fold in both positive and negative *z*-directions to generate gas regions on either side of the copolymer slab.^44,45^ A total of 87 CO_2_ molecules were then inserted into the gas phase, with the number of molecules determined using the ideal-gas relationship based on the volume of the gas region at 300 K and 1 bar, thereby generating the polymer-gas interfacial system for adsorption simulations under ambient conditions. This setup (Fig. S2(b)) allows direct observation of CO_2_ adsorption, surface accumulation, and diffusion into the heterogeneous polymer network. In addition, pressure-dependent simulations were performed by varying the number of CO_2_ molecules in the gas phase, allowing construction of adsorption isotherms and direct correlation between gas pressure and uptake capacity.

#### Bulk copolymer-CO_2_ simulations

To complement the interfacial picture, the bulk-phase configuration containing pre-adsorbed CO_2_ within the polymer matrix was also simulated to examine the microscopic organization, local coordination environments, and structural rearrangements induced by CO_2_ incorporation under dense conditions. The equilibrium CO_2_ loading obtained from the interfacial adsorption simulations was subsequently introduced into the bulk copolymer matrix to construct a fully mixed copolymer-CO_2_ system (Fig. S2(c)). This mixed bulk configuration represents a confined, fully mixed phase where CO_2_ molecules are distributed throughout the polymer network.

#### Simulation protocol

The initial configuration of each system was first energy-minimized using the steepest descent algorithm until the maximum force was below 10 kJ mol^−1^ nm^−1^. Subsequent equilibration protocols depended on the simulation system. For the copolymer-gas interfacial configuration the canonical NVT ensemble was employed to equilibrate the system for 20 ns with a time step of 1.0 fs. Conversely, a three-step CO_2_ procedure was used for the bulk CO_2_ polymer mixed system. The bulk copolymer-CO_2_ systems were equilibrated using the same annealing protocol described for the equilibrated copolymer model, followed by a 50 ns of NPT equilibration at 300 K and 1 bar. Finally, the production MD runs were immediately carried out for 20 ns in the NPT ensemble to obtain reliable results. Temperature control was achieved using the V-rescale thermostat,^73^ with a thermostat coupling time constant (*τ*_t_) of 0.1 ps. Pressure coupling was performed using the Berendsen barostat^74^ during equilibration and the Parrinello–Rahman barostat^75^ during production runs, with a barostat coupling time constant (*τ*_p_) of 2.0 ps. Production trajectories were analysed to determine number and mass density distributions, radial distribution functions (RDFs), CO_2_ adsorption isotherms, and adsorption free energies within the porous copolymer network.

## Results and discussion

### Electronic-structure calculations

To obtain molecular-level insight into the CO_2_ capture, DFT calculations were performed to investigate the interactions between CO_2_ and the representative ion pairs of the 1,2,4-triazolium-based ionic moieties employed in this work. The cations considered are 4-(3-aminopropyl)-1-methyl-1,2,4-triazolium ([aTrz]^+^), 4-(cyanomethyl)-1-methyl-1,2,4-triazolium ([cTrz]^+^), and 4-(2-hydroxyethyl)-1-methyl-1,2,4-triazolium ([eTrz]^+^), combined with bromide ([Br]^−^) and acetate ([Ac]^−^) anions. These ion pairs represent the ionic environments in corresponding porous copolymer networks.

The geometries of all ion pairs (Fig. S3) and DVB were fully optimised at the M06-2X/6-311++G** level of theory. Since the optimised structure and electronic characteristics of DVB remain identical in all six copolymer systems, its DFT analysis is not discussed in the main text and is presented only in the SI. The optimised ion-pair structures were subsequently employed to examine their intrinsic interactions with CO_2_ and to identify the preferred binding sites, interaction strengths, and electronic factors governing CO_2_ adsorption.

#### MESP analysis and prediction of CO_2_ interaction sites

Since CO_2_ behaves as a Lewis acid, it preferentially interacts with electron-rich regions of the molecular framework. To identify these potential binding sites, MESP surfaces were computed at the same level of theory as the geometry optimisations. Regions exhibiting the minimum electrostatic potential (*V*_min_) correspond to areas of highest electron density and therefore denote the most favourable interaction sites for electrophilic species like CO_2_. Consequently, MESP analysis provides a useful first indication of the preferred CO_2_ adsorption sites within the investigated ionic systems.

**Fig. 1:**
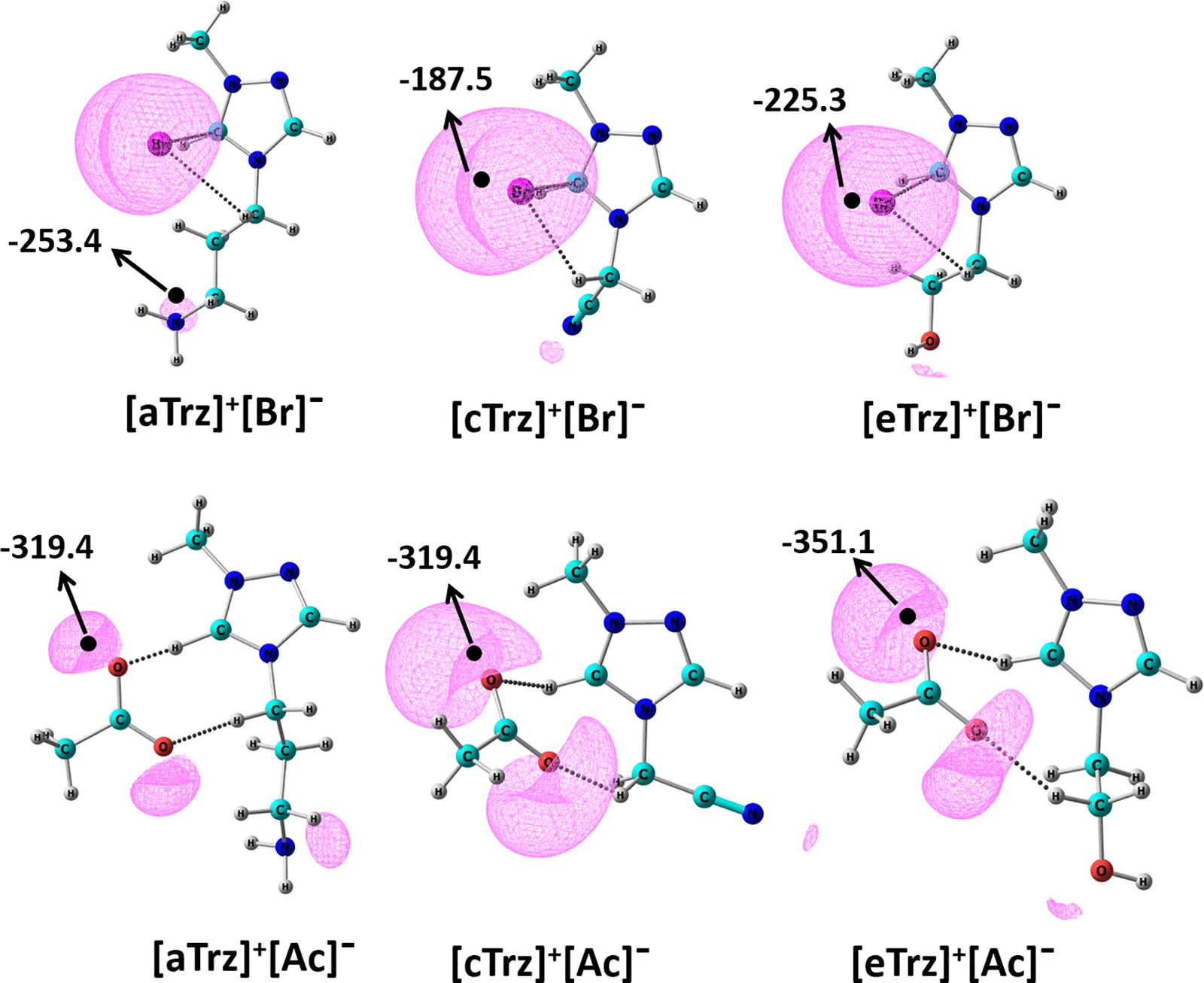
MESP plotted on ILs with the corresponding IL names and their respective *V*_min_ in kJ mol^−1^ calculated at M06-2X/6-311++G** level.

Fig. 1 shows the MESP surfaces and the corresponding *V*_min_ locations for all ion pairs considered in this study, while the MESP map of the divinylbenzene (DVB) cross-linker is provided in the SI (Fig. S4). In most of the cases, the *V*_min_ was observed around the anion indicating that the anionic centre is the primary site for CO_2_ interaction. An exception is observed for the [aTrz]^+^[Br]^−^ ion pair, where the deepest electrostatic potential (*V*_min_) is located on the nitrogen atom of the aminopropyl substituent attached to the triazolium ring. Accordingly, CO_2_ preferentially interacts with this amine nitrogen rather than with the bromide anion.

#### Structure and energetics of CO_2_ binding from DFT calculations

**Fig. 2:**
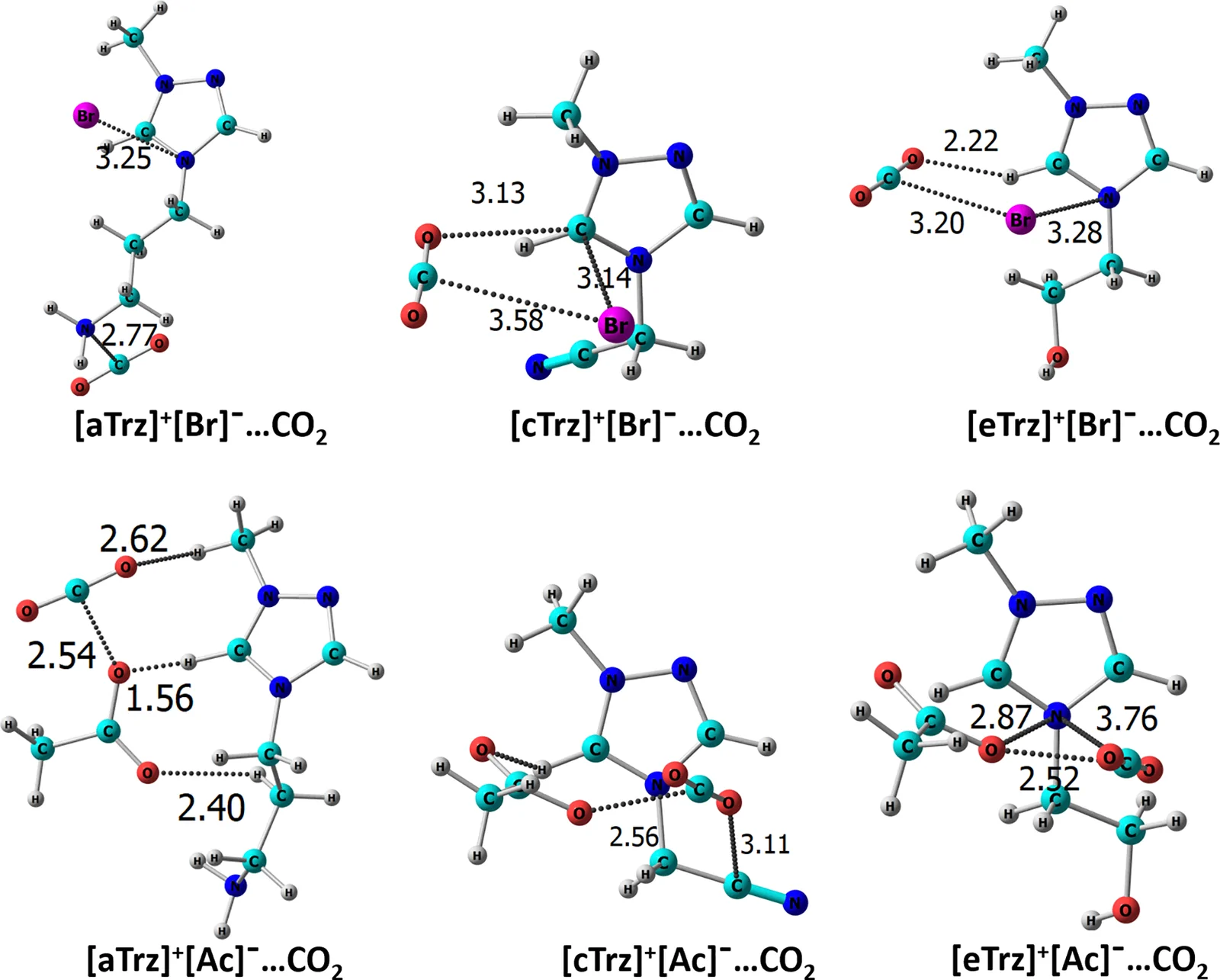
Optimised geometries of the ILs⋯CO_2_ complexes with the corresponding complex names indicated in the figure and their distances of interaction (*d*_int_), in Å, calculated at M06-2X/6-311++G** level.

The preferred CO_2_ binding configurations identified from the MESP maps were subsequently optimised at the M06-2X/6-311++G** level of theory, and their interaction energies (Δ*E*_1_ and Δ*E*_2_) were subsequently evaluated. The optimised geometries are shown in Fig. 2, while the corresponding interaction energies are summarized in Table 1.

**Table 1:** ZPE and BSSE corrected Δ*E*_1_, Δ*E*_2_, (kJ mol^−1^) of IL⋯CO_2_ complexes at M06-2X/6-311++G** level

System	Δ*E*_1_ (kJ mol^−1^)	Δ*E*_2_ (kJ mol^−1^)
([aTrz]^+^[Br]^−^)CO_2_	−418.8	−15.8
([cTrz]^+^[Br]^−^)CO_2_	−470.3	−23.5
([eTrz]^+^[Br]^−^)CO_2_	−434.9	−25.2
([aTrz]^+^[Ac]^−^)CO_2_	−486.7	−19.8
([cTrz]^+^[Ac]^−^)CO_2_	−528.1	−27.7
([eTrz]^+^[Ac]^−^)CO_2_	−475.2	−21.3

Consistent with the MESP predictions, the [aTrz]^+^[Br]^−^ ion pair exhibits its preferred CO_2_ binding site at the amine nitrogen of the aminopropyl substituent rather than at the bromide anion, yielding an interaction energy (Δ*E*_2_) of −15.8 kJ mol^−1^. In contrast, for the remaining five ion pairs, CO_2_ preferentially binds at the electron-rich anionic centres identified from the MESP surfaces.

The interaction energies summarized in Table 1 demonstrate that acetate-containing ion pairs consistently bind CO_2_ more strongly than their bromide counterparts, indicating the superior affinity of the acetate anion toward CO_2_. Among the three functionalized triazolium cations, the [cTrz]^+^ system consistently displays the strongest interactions with CO_2_, followed by [aTrz]^+^ and [eTrz]^+^. Consequently, the [cTrz]^+^[Ac]^−^ ion pair exhibits the most favourable CO_2_ binding among all the systems investigated.

A comparison between the interaction energies and the corresponding MESP minima (Fig. S5) indicates a clear relationship between the magnitude of the electrostatic potential and the strength of CO_2_ binding. This correlation is particularly evident for the bromide-based ion pairs, whereas a less systematic trend is observed for the acetate-containing systems. The weaker correlation in the latter case is likely associated with the more delocalized electron density and the multiple interaction sites available on the acetate anion, resulting in a more distributed CO_2_ binding pattern.

#### QTAIM analysis

**Fig. 3:**
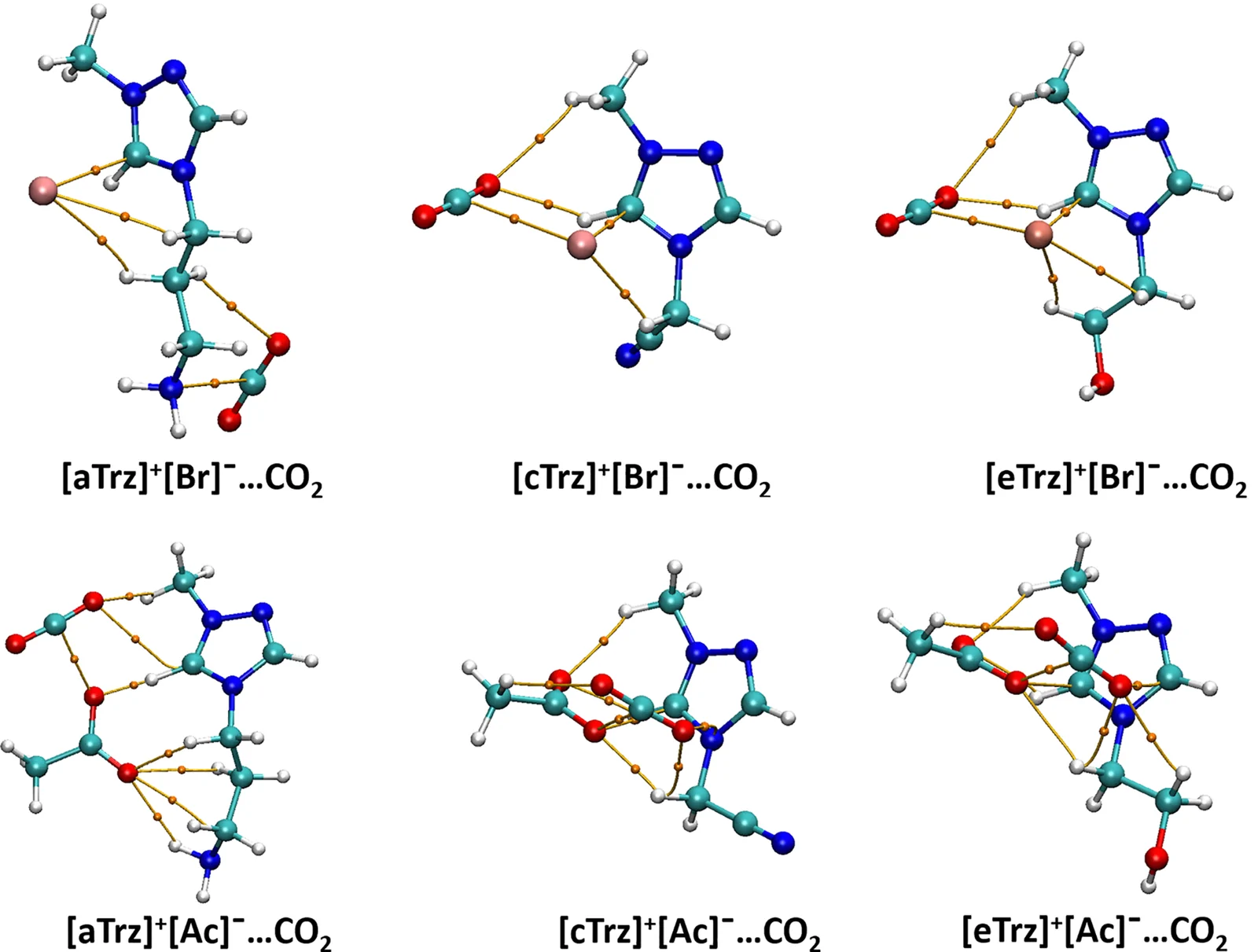
QTAIM plots for IL⋯CO_2_ complexes at the M06-2X/6-311++G** level. The corresponding IL–CO_2_ complex names are indicated in the figure. Orange balls indicate the bcps, and orange lines represent the bond paths of interactions.

The bonding characteristics of the optimised IL⋯CO_2_ complexes were subsequently explored using the QTAIM. Unlike interaction energies, which quantify the overall stability of the complexes, QTAIM provides a topological description of the electron density (*ρ*(*r*)) distribution and enables identification and characterization of the individual intermolecular contacts contributing to CO_2_ adsorption. The molecular graphs obtained from the QTAIM analysis are presented in Fig. 3. According to the Koch and Popelier criterion, closed-shell (noncovalent) interactions are characterized by electron density at the bcp (*ρ*_b_) falls in the range of 0.002–0.040 a.u. together with a positive ∇^2^*ρ*, typically in the range of 0.024–0.139 a.u.^76,77^ These descriptors thus provide a convenient basis for distinguishing the noncovalent interactions responsible for stabilizing the IL⋯CO_2_ complexes.

The molecular graphs reveal that CO_2_ is stabilized through several simultaneous intermolecular contacts rather than a single localized interaction. Multiple bond paths connecting CO_2_ with both the cation and anion are observed in every complex, indicating that the overall stabilization results from the cooperative contribution of individual noncovalent interactions. The properties at bcps can be used to reveal the characteristics of corresponding chemical bonds and interactions, including their nature and strength. Hence to quantify this cumulative effect, the summed electron density (Σ*ρ*_b_), total Laplacian of electron density (Σ∇^2^*ρ*), and the sum of energy density (ΣH*ρ*) at the bcps between the ion-pair and CO_2_ are listed in Table 2.

**Table 2:** The sum of electron density (Σ*ρ*_b_), total Laplacian of electron density (Σ∇^2^*ρ*), and total electron energy density (ΣH*ρ*) at the bcps between the ILs and CO_2_ in atomic units (a.u.) at the M06-2X/6-311++G** level

System	Σ*ρ*_b_	Σ∇^2^*ρ*	ΣH*ρ*
[aTrz]^+^[Br]^−^⋯CO_2_	0.0232	0.0785	0.0020
[cTrz]^+^[Br]^−^⋯CO_2_	0.0298	0.1086	0.0045
[eTrz]^+^[Br]^−^⋯CO_2_	0.0297	0.1081	0.0045
[aTrz]^+^[Ac]^−^⋯CO_2_	0.0338	0.1354	0.0041
[cTrz]^+^[Ac]^−^⋯CO_2_	0.0432	0.1663	0.0048
[eTrz]^+^[Ac]^−^⋯CO_2_	0.0337	0.1284	0.0033

Among the investigated systems, the [aTrz]^+^[Br]^−^⋯CO_2_, complex exhibits a bond path between the carbon atom of CO_2_ and the nitrogen atom of the aminopropyl substituent, with *ρ*_b_ = 0.0154 a.u. and a positive value of ∇^2^*ρ.* These topological characteristics clearly identify the C⋯N contact as a strong noncovalent interaction, in agreement with the MESP prediction and the optimised geometry. The cumulative QTAIM descriptors (Σ*ρ*_b_ and Σ∇^2^*ρ*) also fall within the characteristic range of closed-shell interactions, confirming that CO_2_ binding in this system is dominated by noncovalent forces.

**Fig. 4:**
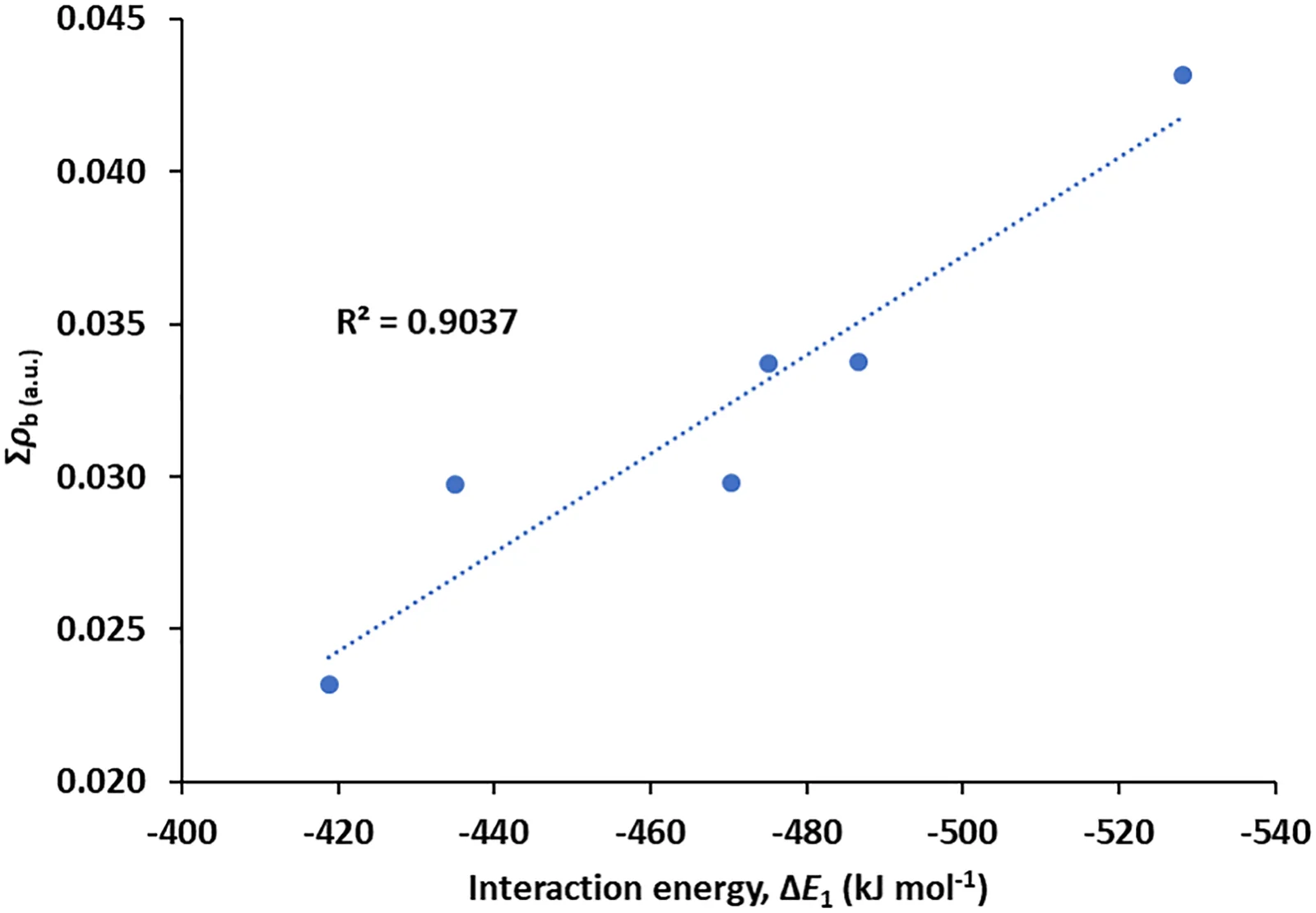
Correlation between Σ*ρ*_b_ in a.u. and Δ*E*_1_ in kJ mol^−1^ for the ILs⋯CO_2_ complexes.

A similar analysis of the remaining complexes leads to the same conclusion. Although the number and arrangement of bond paths differ among the ion pairs, all IL⋯CO_2_ complexes exhibit positive ∇^2^*ρ* values together with *ρ*_b_ values characteristic of noncovalent interactions. Furthermore, the Σ*ρ*_b_ shows an excellent linear correlation with the total interaction energy Δ*E*_1_ (Fig. 4), demonstrating that greater reorganization of the electron density along the bonding regions is stabilizing the complexes.

#### FMO analysis

To further examine the electronic response of the ion pairs upon CO_2_ adsorption, FMO analysis was performed on the optimised IL⋯CO_2_ complexes on the ILs. The energies and spatial distributions of the highest occupied molecular orbital (HOMO) and the lowest unoccupied molecular orbital (LUMO) provide useful information on electron distribution and the electronic stability of the interacting systems.^78,79^ The gap between these FMOs, known as the frontier orbital energy gap, (Δ*E* = *E*_LUMO_ − *E*_HOMO_) was also evaluated, where a larger gap generally reflects greater electronic stability.

**Fig. 5:**
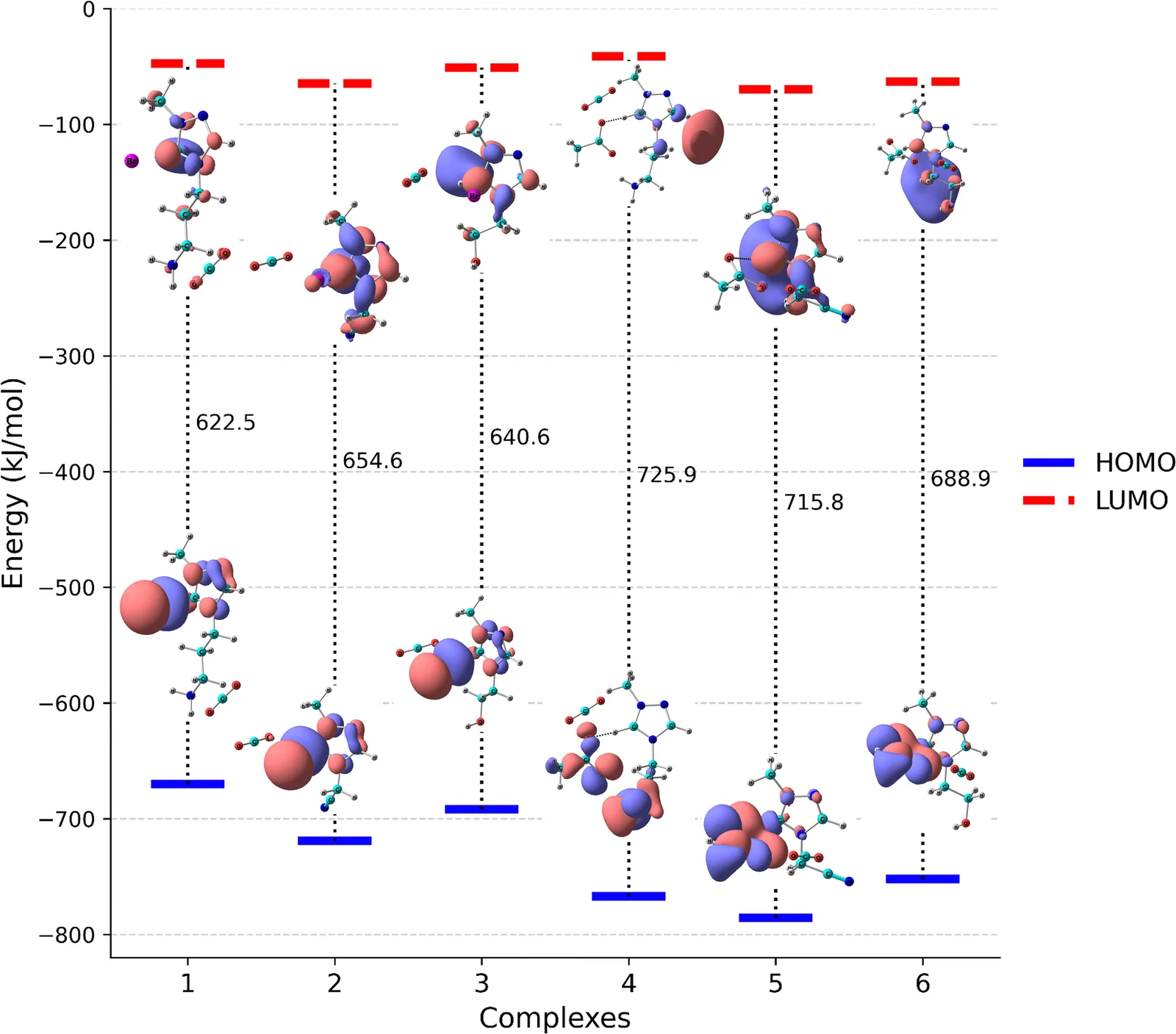
HOMO, LUMO (isosurface = 0.03 a.u.) of various ILs⋯CO_2_ systems. Pink and blue colour distributions represent positive and negative phases in the molecular orbital wave function, respectively. The numbers on the dotted lines represent the values of the HOMO–LUMO gap in kJ mol^−1^.

Fig. 5 presents FMO diagrams and their energy for the six ILs⋯CO_2_ systems in this study. Noticeably, the electron cloud of the HOMO is mainly distributed in the anion part for each ILs⋯CO_2_ system, suggesting that the anion has the highest electron density and is more likely to provide electrons in a chemical reaction and thus can serve as an electron donor for the reaction. The LUMO electron cloud is predominantly localized in the cation, mostly around the atoms close to the anion, revealing that these parts can act as electron acceptors in a reaction.

The FMOs of the ILs prior to the presence of CO_2_ were also calculated and displayed in Fig. S6. Comparison with the isolated ion pairs shows that CO_2_ adsorption produces only minor changes in the HOMO distribution, whereas the LUMO becomes more localized following complex formation. This behaviour suggests a redistribution of electron density within the ion pair upon interaction with CO_2_ while largely preserving the electronic characteristics of the individual ions. In addition, most IL⋯CO_2_ complexes exhibit a slight increase in the HOMO–LUMO energy gap relative to the corresponding isolated ion pairs, indicating a modest enhancement in electronic stability after CO_2_ adsorption.

It should be noted that although frontier molecular orbitals provide valuable information regarding electronic structure and reactivity, CO_2_ adsorption is governed by the combined influence of several factors, including electrostatic interactions, charge redistribution, steric effects, and the availability of favourable binding sites. Consequently, the FMO analysis should be interpreted together with the interaction energy, MESP, and QTAIM results to obtain a comprehensive understanding of the adsorption mechanism.

Overall, the DFT results provide a clear molecular-level picture of CO_2_ binding within the triazolium-based systems. MESP and interaction-energy analyses identify the preferred CO_2_ binding sites and show generally stronger CO_2_ interactions for acetate-containing systems, with [cTrz]^+^[Ac]^−^ exhibiting the most favourable binding. QTAIM analysis confirms that these interactions are mainly noncovalent and involve multiple sites on both the cation and anion. The FMO results further indicate complementary electronic roles of the anionic and cationic components. These findings demonstrate that both counterion and cation structure influence CO_2_ binding and provide a molecular basis for interpreting the adsorption and transport behaviour observed in the subsequent MD simulations.

### Molecular dynamics simulations

While the DFT calculations provide molecular-level information on the intrinsic affinity of individual ion pairs toward CO_2_, they do not capture the collective adsorption and transport processes occurring within the porous polymer framework. To address these aspects, all-atom MD simulations were performed on the corresponding cross-linked porous copolymers constructed from the six triazolium-based ionic units and DVB cross-linkers, as described in the Computational methods section. These copolymer systems are designated as CP1–CP6, and their compositions and simulation details are summarized in Table S1 and Fig. S1 and S2 (SI).

Unlike previously reported ionic liquid systems where CO_2_ absorption occurs in a homogeneous liquid environment, the present study investigates adsorption in a heterogeneous cross-linked porous polymer, where confinement and functional group distribution jointly govern CO_2_ uptake and transport. An equilibrated amorphous copolymer matrix was first generated for each system and subsequently used as the reference configuration for constructing the CO_2_ adsorption and bulk copolymer-CO_2_ simulation models. These models were employed to investigate adsorption behaviour, molecular transport, and equilibrium distribution of CO_2_ within these porous polymer networks. The equilibrium density and structural characteristics of the pristine copolymer matrices are summarized in Table S1. The corresponding copolymer materials have also been experimentally characterized as mesoporous, establishing the porous nature of these copolymer networks and supporting their suitability for CO_2_ adsorption and transport.^33^

#### CO_2_ adsorption dynamics

The adsorption kinetics of CO_2_ were first examined by monitoring the number of molecules adsorbed into the porous copolymer as a function of simulation time (Fig. S7). A rapid uptake of CO_2_ is observed during the initial stage of the simulation, with the adsorption process approaching equilibrium within approximately 2 ns. After equilibration, adsorption capacities were obtained by averaging over the stabilized portion of the trajectories to account for thermal fluctuations.

**Fig. 6:**
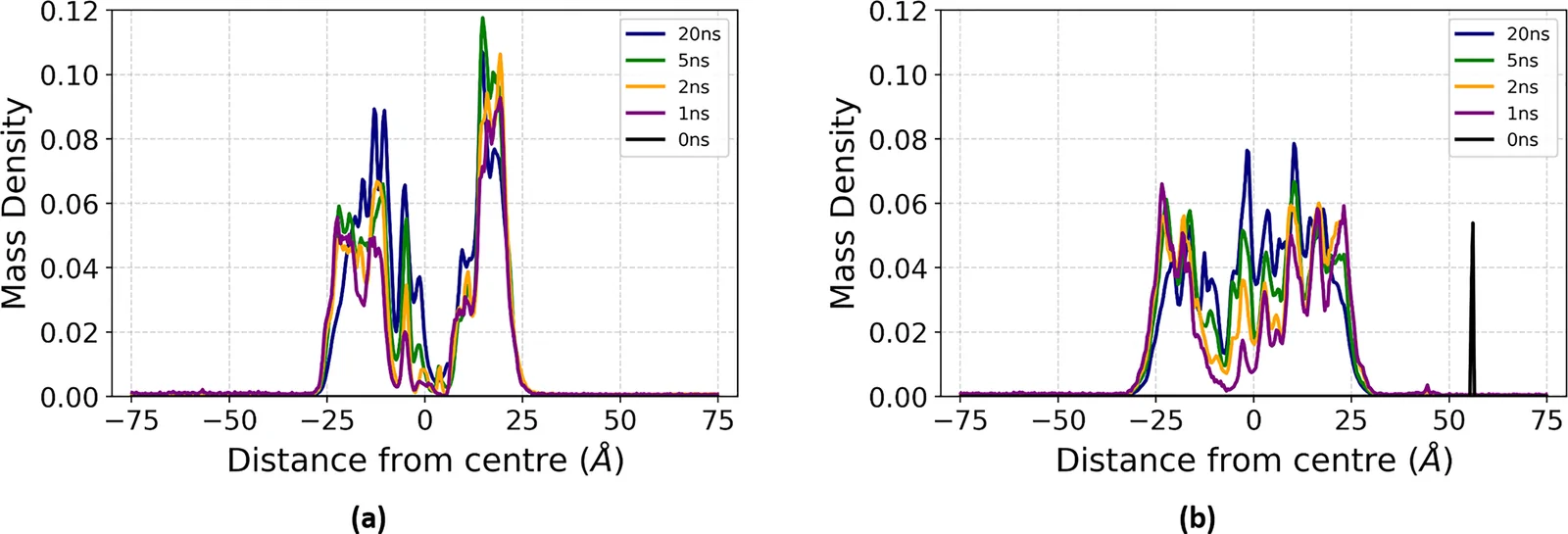
Mass density (g cm^−3^) profiles of CO_2_ in the *z*-direction of the copolymers (a) CP1 and (b) CP5 at 0 ns, 1 ns, 2 ns, 5 ns, and 20 ns. At 0 ns, CO_2_ is initially confined to the gas-phase regions, resulting in negligible CO_2_ density within the copolymer slab.

**Fig. 7:**
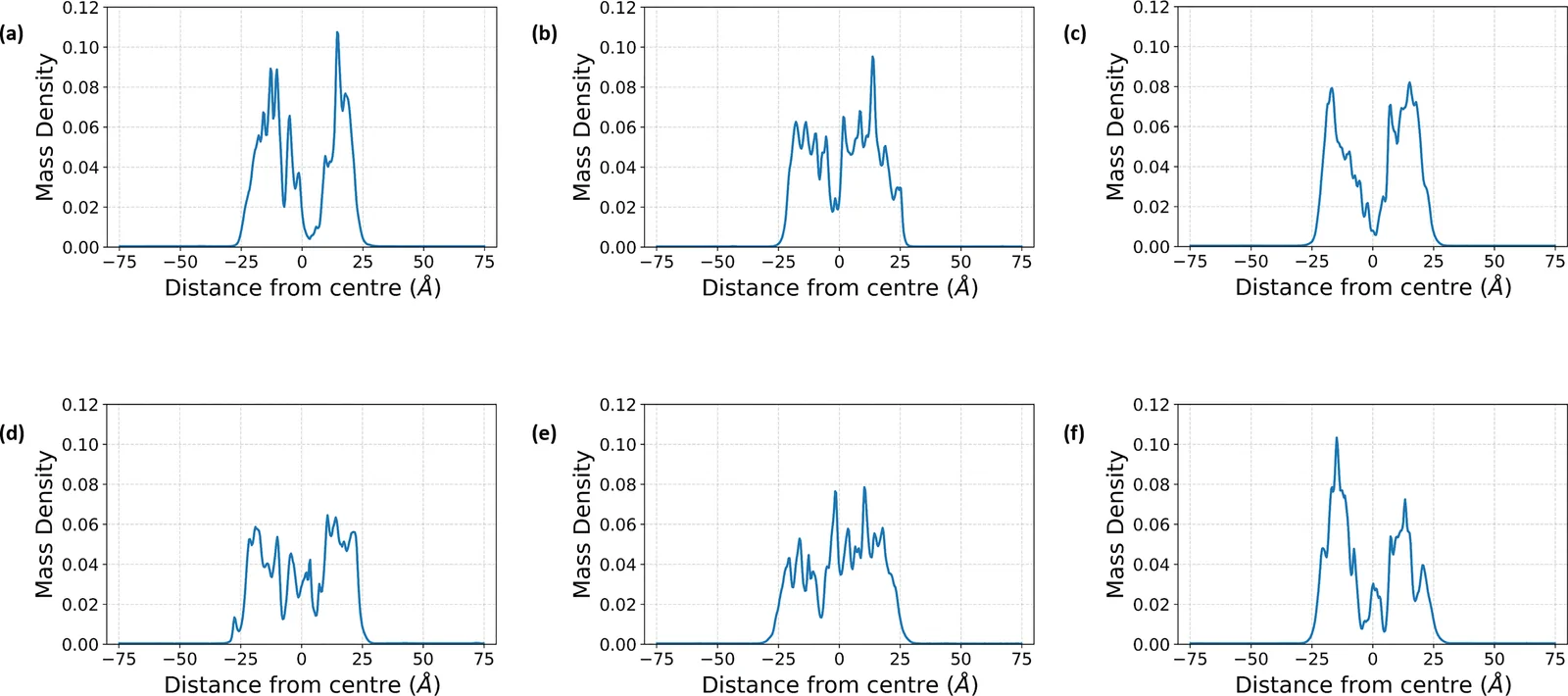
Mass density (g cm^−3^) distributions of CO_2_ in the *z*-direction of copolymer systems. (a) CP1 ([aTrz]^+^[Br]^−^) (b) (CP2[cTrz]^+^[Br]^−^), (c) CP3 ([eTrz]^+^[Br]^−^), (d) CP4 ([aTrz]^+^[Ac]^−^) (e) CP5 ([cTrz]^+^[Ac]^−^) and (f) CP6([eTrz]^+^[Ac]^−^) [*P* = 1 atm, *T* = 298.15 K].

The evolution of CO_2_ mass-density profiles along the *z*-direction was monitored at selected simulation times to visualize the progressive adsorption of CO_2_ into the copolymer. The CO_2_ mass density in each *z*-bin was calculated by dividing the total mass of CO_2_ molecules within the bin by its corresponding volume. The profiles in Fig. 6 represent the CO_2_ mass-density averaged from start to the indicated simulation times, whereas the equilibrium profiles in Fig. 7 were obtained by averaging over the equilibrated portion (2–20 ns) of the trajectory. At the beginning of the simulation (0 ns), CO_2_ molecules are confined to the gas phase, with only a negligible population near the copolymer surface; consequently, the CO_2_ mass density within the copolymer slab is essentially zero. As the simulation proceeds, CO_2_ rapidly accumulates at the copolymer-gas interface, producing a pronounced interfacial density peak within 1 ns. With continued simulation time, CO_2_ progressively diffuses from the interface into the internal porous network, accompanied by a gradual decrease in interfacial concentration and a corresponding increase in bulk loading. By approximately 2 ns, noticeable penetration into the copolymer is already evident, while at longer times (5–20 ns), the density maximum shifts progressively toward the interior of the copolymer matrix, indicating continued redistribution within the pore structure These observations demonstrate that CO_2_ adsorption is a dynamic multistep process involving initial surface adsorption followed by diffusion into the interconnected porous structure until equilibrium is attained.

Once adsorption equilibrium is established, CO_2_ density along the *z*-direction can be compared among the different copolymer systems (Fig. 7). In all cases, significant CO_2_ accumulation is observed near the copolymer-gas interface, confirming that adsorption initially occurs at the external surface before diffusion into the polymer matrix. However, significant CO_2_ populations are also present throughout the interior of the copolymers, indicating efficient pore accessibility and internal diffusion of CO_2_ within the porous copolymers.

Distinct differences are observed in the CO_2_ density distributions depending on the ionic composition and molecular structure of the copolymer. Copolymers containing Br^−^ mostly exhibit pronounced interfacial density maxima (Fig. 7a–c), accompanied by a pronounced decrease in CO_2_ density toward the centre of the polymer slab, suggesting preferential interfacial adsorption. In contrast, the corresponding Ac^−^ systems (Fig. 7d–f) display broader CO_2_ distributions with appreciable density within the polymer interior, suggesting enhanced CO_2_ penetration and more homogeneous distribution throughout the polymer matrix. However, noticeable variations are observed among the individual profiles, as illustrated by Fig. 7b and d, indicating that both the counter anion and cationic structure influence the spatial distribution of CO_2_ within the polymer. The greater CO_2_ penetration observed for the acetate-containing copolymers is consistent with previous reports on PILs showing enhanced CO_2_ affinity in the presence of acetate counter anions.^80^

The adsorption of CO_2_ in these copolymers therefore involves both interfacial accumulation and penetration of CO_2_ molecules into the polymer network. The triazolium substituent on the copolymer also influences the adsorption behaviour. Among the three cations investigated, copolymers containing [cTrz]^+^ exhibit the most extensive CO_2_ penetration into the polymer matrix, including in the presence of bromide counter-ions. In contrast, systems based on [eTrz]^+^ show comparatively sharp interfacial density maxima and lower CO_2_ concentrations near the centre of the polymer, indicating efficient initial adsorption but relatively limited diffusion into the interior. Overall, the simulated adsorption behaviour suggests the cation-dependent trend: [cTrz]^+^ > [eTrz]^+^ > [aTrz]^+^, in terms of CO_2_ uptake within the porous copolymers. This trend agrees well with the interaction energies obtained from the DFT calculations, indicating that intrinsic IL–CO_2_ interaction strengths govern the observed CO_2_ uptake and provide a link between molecular-level interactions and CO_2_ adsorption at the polymer scale.

### Pressure-dependent CO_2_ adsorption

**Fig. 8:**
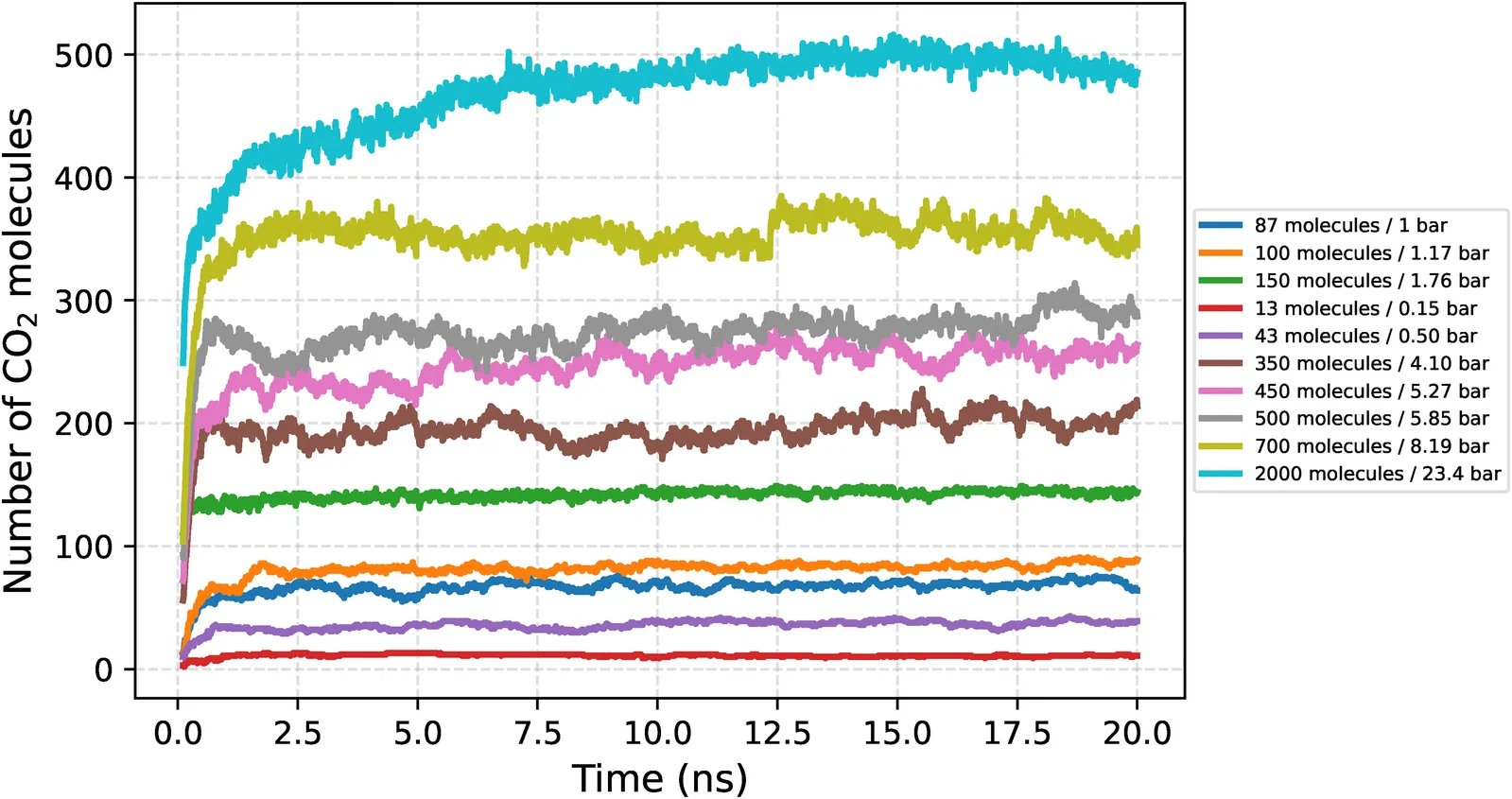
Evolution of the number of CO_2_ being adsorbed in [aTrz]^+^[Br]^−^based copolymers at various CO_2_ gas phase pressure conditions.

The influence of gas-phase pressure on CO_2_ uptake was further investigated by performing adsorption simulations over a range of CO_2_ pressures. Fig. 8 presents the time-dependent uptake profile for the representative copolymer CP1, where different pressures were generated by varying the number of CO_2_ molecules introduced into the gas phase. Similar adsorption behaviour was obtained for the remaining copolymers.

For all pressure conditions, CO_2_ adsorption proceeds rapidly during the initial stage of the simulation, with the uptake approaching equilibrium within approximately 2 ns. This rapid initial adsorption reflects the favourable interactions between CO_2_ and the ionic framework together with the rapid transport of CO_2_ molecules toward the accessible adsorption sites. Following the initial uptake, the CO_2_ loading fluctuates around an equilibrium value. The fluctuations become somewhat more pronounced at higher pressures, consistent with the larger number of CO_2_ molecules and their continued redistribution within the interfacial and porous regions.

As the gas-phase pressure increases, the equilibrium CO_2_ loading increases progressively. At the highest simulated pressure (≈23.4 bar) approximately 500 CO_2_ molecules are present in the adsorbed population. The corresponding adsorption profiles show Type I-like behaviour, characterized by increasing CO_2_ uptake with pressure and a progressively reduced slope at higher pressures. Overall, the results demonstrate that the porous triazolium-based copolymers continue to accommodate increasing amounts of CO_2_ over the investigated broad pressure range while maintaining rapid adsorption kinetics, highlighting its potential for efficient CO_2_ capture applications.

#### Henry's constant and adsorption free energy

The equilibrium adsorption data obtained in the low-pressure regime were further analysed using Henry's law to evaluate the intrinsic affinity of the copolymer toward CO_2_. The corresponding Henry-law analysis is presented in Fig. S8, provides a quantitative measure of the CO_2_ adsorption under dilute conditions. The calculated Henry constant corresponds to a CO_2_ uptake of 1.28 mmol g^−1^ bar^−1^, indicating substantial affinity of the porous copolymer for CO_2_, which is in agreement with that observed for similar copolymer systems, experimentally characterized as mesoporous materials, supporting their suitability for CO_2_ adsorption and transport.^33^ Since Henry's law is applicable within the dilute adsorption regime, this analysis provides an appropriate description of the initial CO_2_ adsorption behavior prior to significant occupation of the available adsorption environments.

The thermodynamic favourability of CO_2_ adsorption was further evaluated from the MD simulations by calculating the adsorption free energy (Δ*G*_ads_) using,^81^

5 Δ*G*_ads_ = −RT ln(*C*_ads_/*C*_gas_)

where *C*_ads_ and *C*_gas_ denote the concentrations of adsorbed and gas-phase CO_2_, respectively at *T* = 300 K. The calculated adsorption free energies (Table S2) are negative over the entire investigated pressure range, indicating thermodynamically favourable CO_2_ adsorption under simulated conditions. At ambient conditions (300 K and 1 bar), Δ*G*_ads_ is −12.2 kJ mol^−1^, demonstrating favourable physisorption arising from attractive interactions between CO_2_ and the ionic copolymer framework.

As the gas pressure increases, the adsorption free energy becomes progressively less negative (Fig. S9), indicating a gradual decrease in the thermodynamic driving force for adsorption. This behaviour can be associated with the preferential occupation of more favourable adsorption environments at lower CO_2_ loading, followed by adsorption at progressively weaker sites as the polymer pores become increasingly occupied. At higher pressures, additional CO_2_⋯CO_2_ interactions (crowding effects) within the confined pore space may contribute to the reduced thermodynamic driving force for adsorption. The pressure-dependent evolution of Δ*G*_ads_ is consistent with the uptake profiles in Fig. 8, where rapid initial adsorption is followed by a slower approach toward equilibrium at higher loading. Nevertheless, the negative values of Δ*G*_ads_ throughout the investigated pressure range, indicate that CO_2_ adsorption remains thermodynamically favourable under the simulated conditions.

The adsorption free energies calculated for the remaining five copolymers at 1 bar (Table S3) are likewise negative and fall within the range typically reported for moderately interacting porous adsorbents,^82–84^ confirming the spontaneous nature of CO_2_ adsorption across all six triazolium-based copolymers. Moreover, the moderate magnitude of Δ*G*_abs_ indicates a favourable balance between adsorption strength and regenerability, providing sufficiently strong interactions for efficient CO_2_ capture while avoiding excessively strong binding that would hinder desorption. These characteristics make the investigated copolymers promising candidates for pressure-swing adsorption application processes.

#### RDF analysis of CO_2_ adsorption

**Fig. 9:**
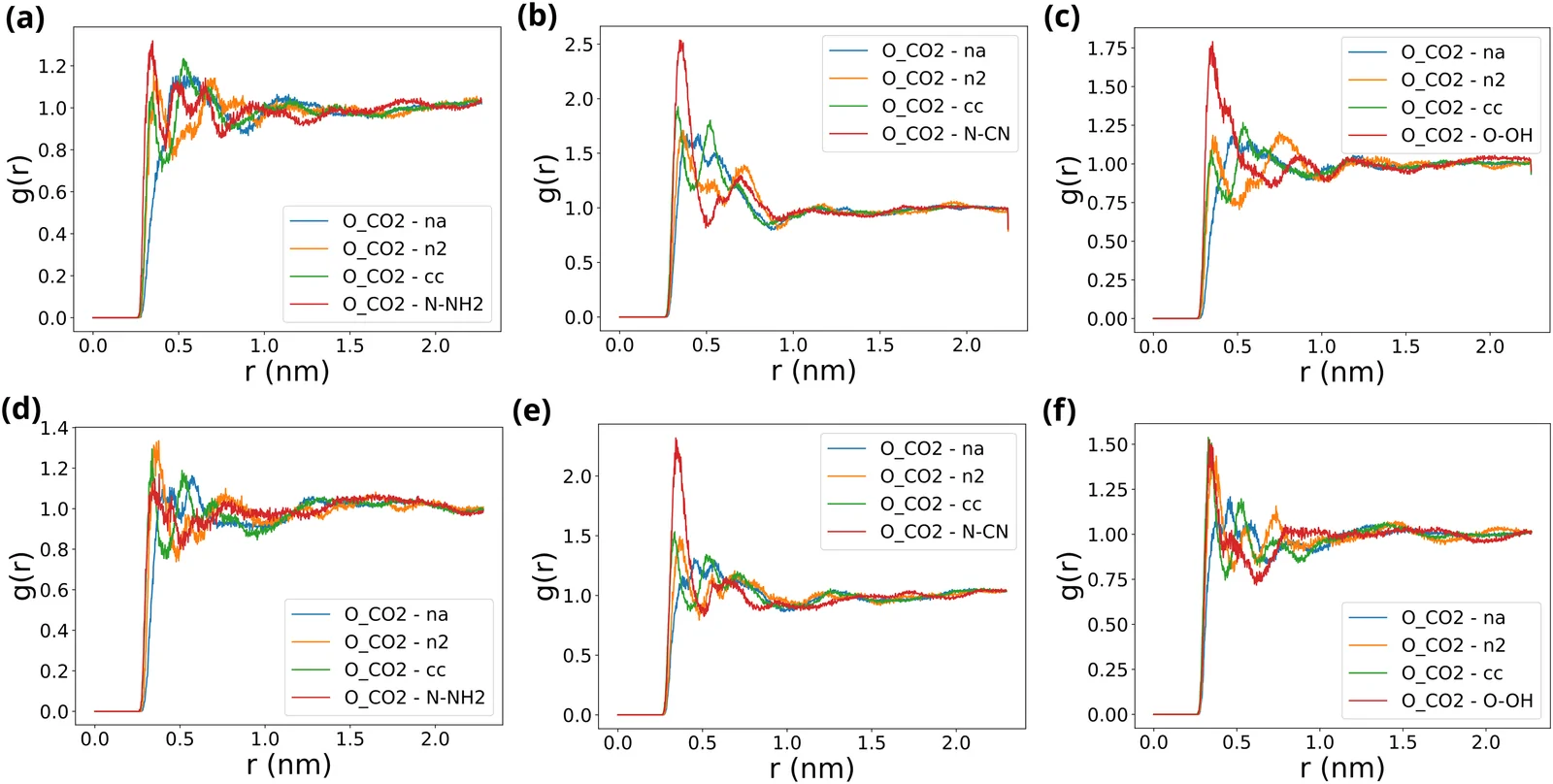
RDF profiles between CO_2_ oxygen atoms and selected atomic centres on the triazolium ring and corresponding functional groups of the copolymers: (a) CP1, (b) CP2, (c) CP3, (d) CP4, (e) CP5, and (f) CP6. The specific CO_2_-atom pairs considered for each copolymer are indicated in the respective legends.

Bulk-phase configurations derived from the average CO_2_ loading in the copolymer-gas interfacial simulations were used for structural analysis. Radial distribution functions (RDF, *g*(*r*)) were computed to characterize the local coordination environment of CO_2_ within the polymer matrix. The RDF profiles between CO_2_ oxygen atoms and selected atomic centres on the triazolium functional framework of the copolymer are presented in Fig. 9, while the corresponding atom labels are provided in Fig. S10. The present RDF analysis focuses on CO_2_ interactions with polymer-bound functional groups; although CO_2_-anion correlations, known to contribute to adsorption in ionic systems, are not explicitly discussed here in order to isolate the role of the covalently bound functional architecture.

In general, CO_2_ framework interactions exhibit relatively broad and low-intensity first coordination peaks, indicating weak and non-specific interactions with the polymer backbone. This suggests that the hydrocarbon framework primarily contributes through steric confinement rather than direct binding. In contrast, pronounced first peaks are observed for CO_2_ interactions with heteroatom-containing functional sites on the triazolium units, particularly the –NH_2_, –CN, and –OH groups. The most intense peak appears at approximately 0.3–0.5 nm, suggesting a high probability of CO_2_ localization near these polar sites. The sharpness of these peaks indicates pronounced local ordering and preferential localization of CO_2_ near these polar sites, consistent with the favourable interactions identified by the DFT calculations. Additional nitrogen sites on the triazolium ring also exhibit noticeable but weaker correlations in a similar radial range, suggesting cooperative contributions from multiple polar centres within the polymer matrix to CO_2_ stabilization, with varying strengths. In addition to the prominent first peaks at shorter distances, several RDF profiles exhibit secondary maxima at larger intermolecular distances. These features represent additional spatial correlations between CO_2_ and the selected polymer atomic centres, arising from the local structural arrangement of neighbouring functional groups and successive structural shells within the polymer framework. The presence of these secondary features reflects the heterogeneous local environment experienced by CO_2_ within the copolymer. At distances beyond ∼1.0–1.2 nm, all RDF curves converge to unity, confirming that these interactions are short-ranged and localized around these functional sites. Overall, the RDF results confirm that CO_2_ preferentially interacts with polar heteroatom sites on the DVB-triazolium framework, while the hydrocarbon backbone primarily provides structural confinement and pore architecture. These findings are consistent with the DFT results, indicating that the molecular-level interactions predicted by DFT are retained within the polymer environment, with CO_2_ structuring localized around the functionalized regions.

To further assess CO_2_ mobility within this heterogeneous polymer environment, the CO_2_ self-diffusion coefficients were calculated from the mean-square displacement (MSD) of pre-adsorbed CO_2_ molecules in the bulk copolymer-CO_2_ simulations; the calculated values are provided in Table S4. These coefficients characterize the translational mobility of CO_2_ after adsorption and complement the RDF and density-profile analyses by providing insight into CO_2_ transport within the polymer network. Overall, the MD simulations establish that CO_2_ capture arises from a cooperative interplay of preferential interactions at polar sites, penetration into the polymer network, and confinement within the cross-linked porous architecture.

## Conclusions

The combined DFT and MD results demonstrate that the CO_2_ capture by the 1,2,4-triazolium based ILs and their porous copolymers is governed by a synergy between electronic structure effects and polymer-scale transport phenomena. At the molecular level, CO_2_ adsorption is primarily driven by noncovalent interactions with anionic sites, while the functional groups attached to the triazolium cations play a crucial role in modulating adsorption strength. In particular, the 3-aminopropyl group on the 1,2,4-triazolium unit enhances CO2 affinity by providing additional electron-rich interaction sites. DFT-based MESP and QTAIM analyses consistently identify preferential CO_2_ interaction regions and confirm the importance of specific functional group environments through bond critical point analysis. FMO analysis further disclosed that CO_2_ adsorption induces electronic redistribution within the ionic systems, leading to additional stabilization through polarization and charge delocalization effects. Collectively, these findings highlight that CO_2_ adsorption is governed not only by electrostatic interactions but also by cooperative electronic effects arising from the local chemical environment.

MD simulations complement this molecular-level picture by revealing the role of polymer architecture of ILs and DVB in governing adsorption and transport behaviour. CO_2_ molecules exhibit rapid migration from the gas phase into the polymer matrix *via* interfacial accumulation followed by diffusion into internal pores, demonstrating that adsorption is strongly influenced by both accessible free volume and functional site distribution. RDF analysis further confirms preferential CO_2_ localization near polar N- and O-containing centres within the triazolium-based framework, consistent with the trends observed from electronic structure calculations. The thermodynamic parameters derived from MD simulations, including adsorption free energies, indicate spontaneous CO_2_ uptake across all systems and show qualitative agreement with trends observed in related porous polymeric materials. These results establish a clear structure–property relationship connecting functional group chemistry, electronic structure, and macroscopic CO_2_ adsorption behaviour.

Overall, this study demonstrates that integrating DFT and MD simulations provides a comprehensive framework for understanding CO_2_ capture in ionic polymeric materials. While DFT elucidates the fundamental interaction mechanisms, MD captures the dynamic adsorption and transport processes within realistic porous environments. These combined insights provide useful guidelines for the rational design of next-generation triazolium-based porous materials for efficient CO_2_ capture applications. Future work may extend this framework to reactive systems to explore chemisorption and CO_2_ conversion pathways.

## Author contributions

Sebastian Anila: conceptualization, methodology, investigation, formal analysis, validation, visualization, data curation, writing – original draft, review and editing. Jiayin Yuan: project administration, funding acquisition, resources, supervision, writing – review and editing. Alexander Lyubartsev: methodology, software, resources, supervision, formal analysis, writing – review and editing.

## Conflicts of interest

There are no conflicts to declare.

## Supplementary Material

CP-028-D6CP02655A-s001

## Data Availability

The data supporting this article, including additional figures, tables, and Cartesian coordinates of the optimised structures, are provided in the supplementary Information (SI). See DOI: https://doi.org/10.1039/d6cp02655a.
