# Supplementary material for: Multiscale investigation of CO2 capture in 1,2,4-triazolium-based ionic liquids and porous copolymers using density functional theory and molecular dynamics
Source: Phys Chem Chem Phys. 2026 Sep 4;28(37):23075–87. doi: 10.1039/d6cp02655a (PMC13563441; doi:10.1039/d6cp02655a)
Supplement: Supplementary file 1 [file CP-028-D6CP02655A-s001.pdf]

Supplementary Information (SI) for Physical Chemistry Chemical Physics.
This journal is © the Owner Societies 2026

# Supporting Information

## Multiscale Investigation of $CO_2$ Capture in 1,2,4-Triazolium-Based Ionic Liquids and Porous Copolymers Using Density Functional Theory and Molecular Dynamics

Sebastian Anila.[a*], Jiayin Yuan[a], and Alexander P. Lyubartsev[a*]

[a] *Department of Chemistry, Stockholm University, S-10691 Stockholm, Sweden.*

*Email: anilasebastian25@gmail.com and alexander.lyubartsev@su.se

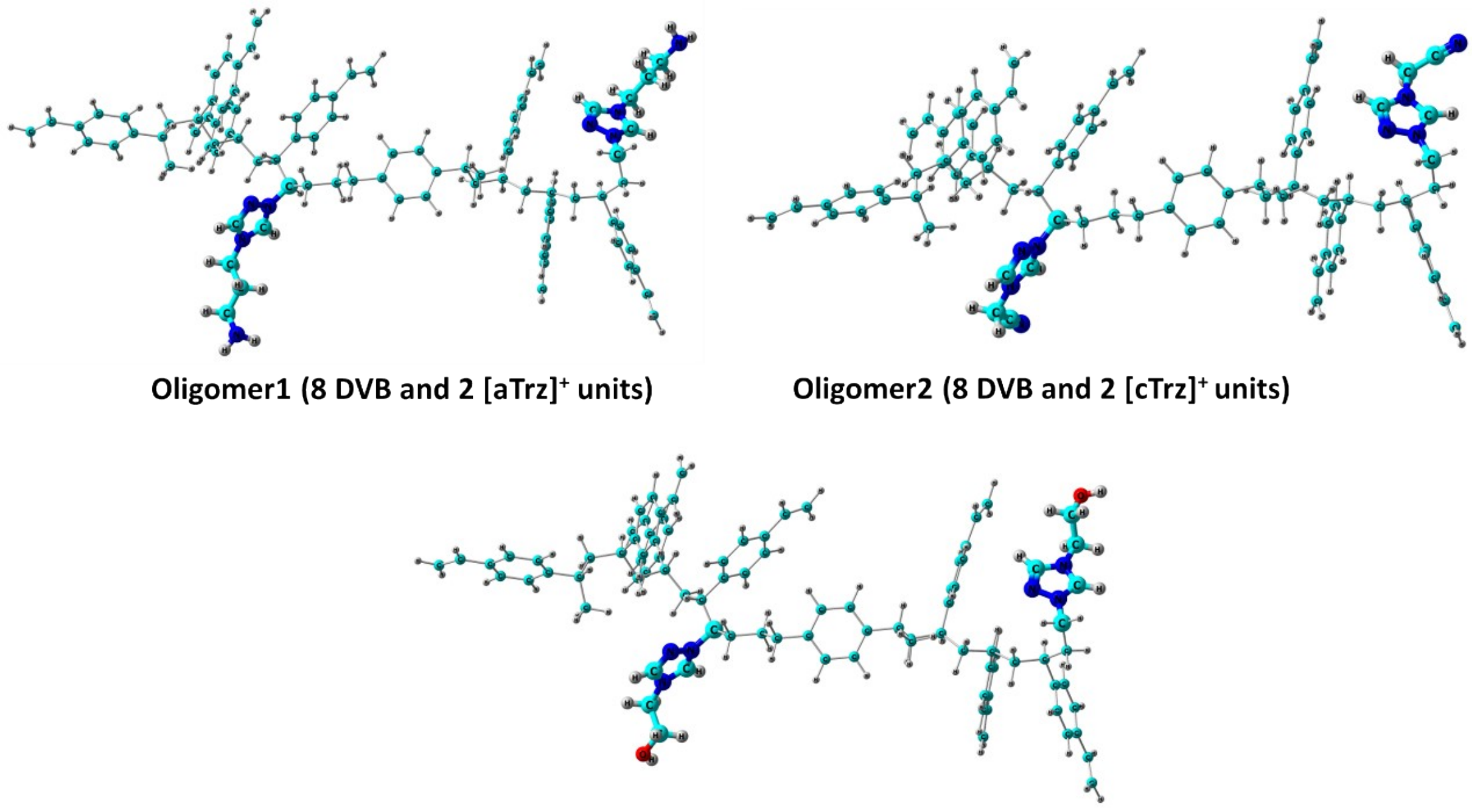

**Oligomer1 (8 DVB and 2 [aTrz]+ units)** **Oligomer2 (8 DVB and 2 [cTrz]+ units)**

**Oligomer3 (8 DVB and 2 [eTrz]+ units)**

**Figure S1.** Optimized geometries of the oligomers (decamers), representing the copolymer backbone, at the B3LYP/6-31G* level. The element-based colours are used, C: green; H: grey; N: blue; and O: red.

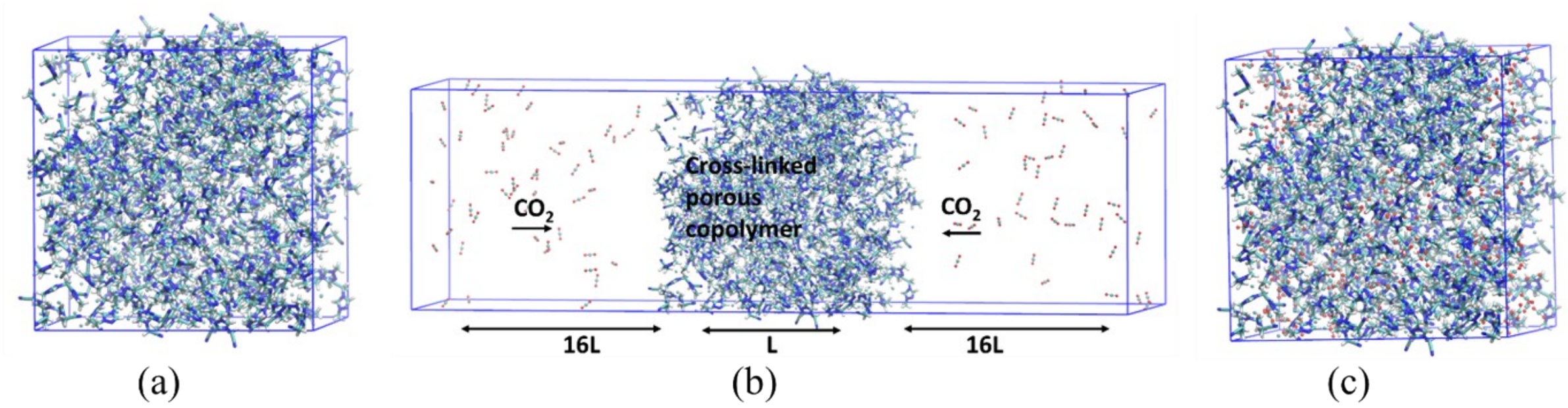


**Figure S2.** MD simulation systems for (a) equilibrated copolymer model, (b) copolymer-gas interfacial system (L=4.8 nm and 16L= 76.8 nm), and (c) bulk copolymer-$CO_2$ system. Colour code: green, blue, red, and grey for C, N, O, and H atoms, respectively

**Table S1**. The composition and density of the equilibrated copolymer models (4.8 x 4.8 x 4.8 $nm^3$).

| System | Oligomer | Cations | Anions | Density ($g/cm^3$) |
|---|---|---|---|---|
| CP1 | Oligomer1 | $[aTrz]^+$ | $[Br]^-$ | 1.12 |
| CP2 | Oligomer2 | $[cTrz]^+$ | $[Br]^-$ | 1.12 |
| CP3 | Oligomer3 | $[eTrz]^+$ | $[Br]^-$ | 1.13 |
| CP4 | Oligomer1 | $[aTrz]^+$ | $[Ac]^-$ | 1.02 |
| CP5 | Oligomer2 | $[cTrz]^+$ | $[Ac]^-$ | 0.92 |
| CP6 | Oligomer3 | $[eTrz]^+$ | $[Ac]^-$ | 1.05 |

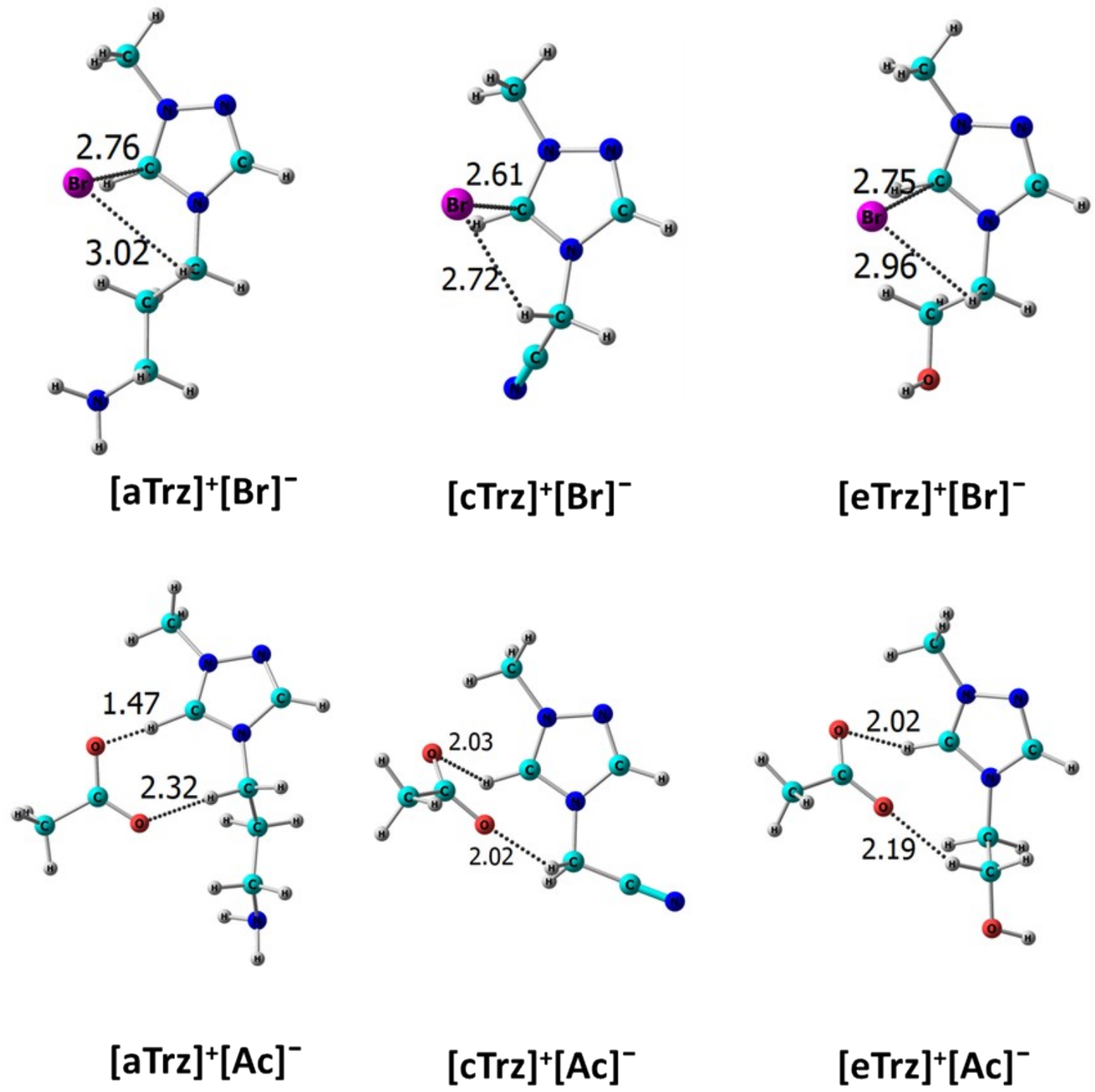


**Figure S3**. Optimized geometries of ion-pair complexes with their distance of interaction ($d_{int}$) in Å at the M06-2X/6-311++G** level.

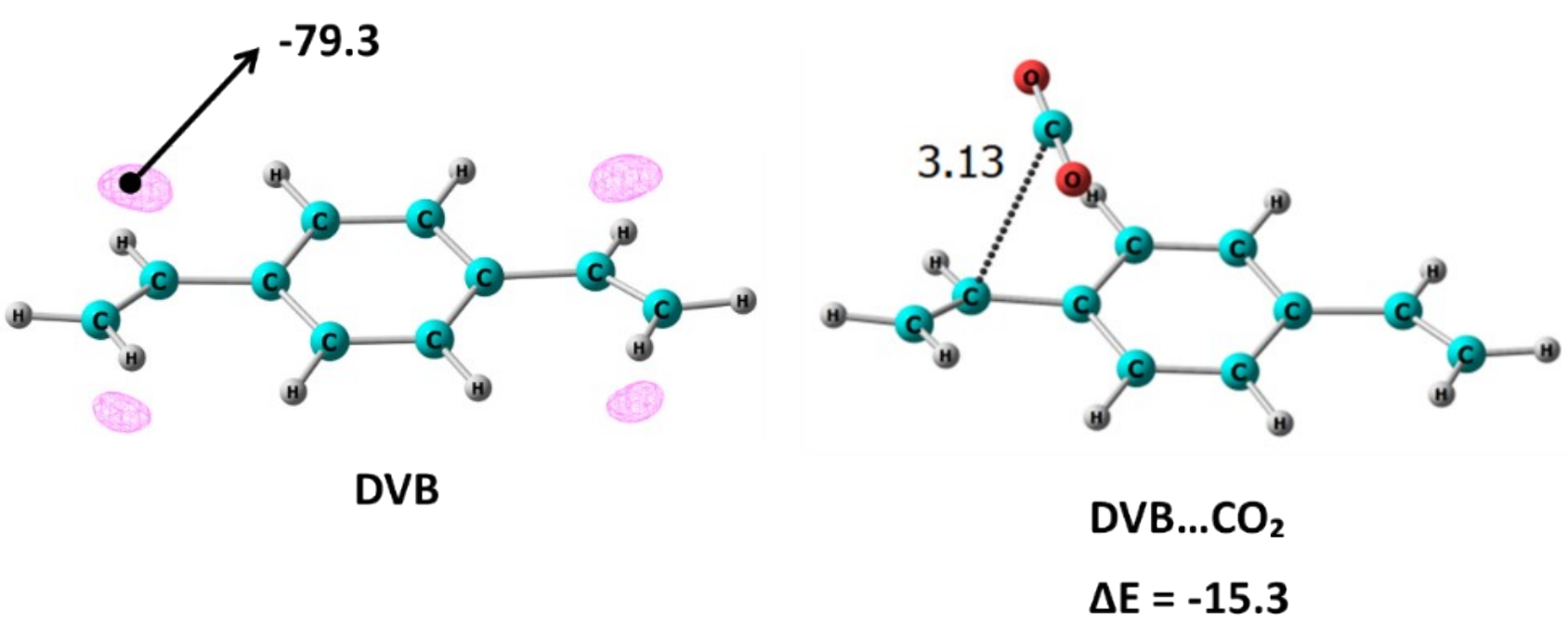


**Figure S4**. Optimized geometries of DVB and DVB…$CO_2$ with the $V_{min}$ (kJ/mol) and $d_{int}$ (Å) at the M06-2X/6-311++G** level.

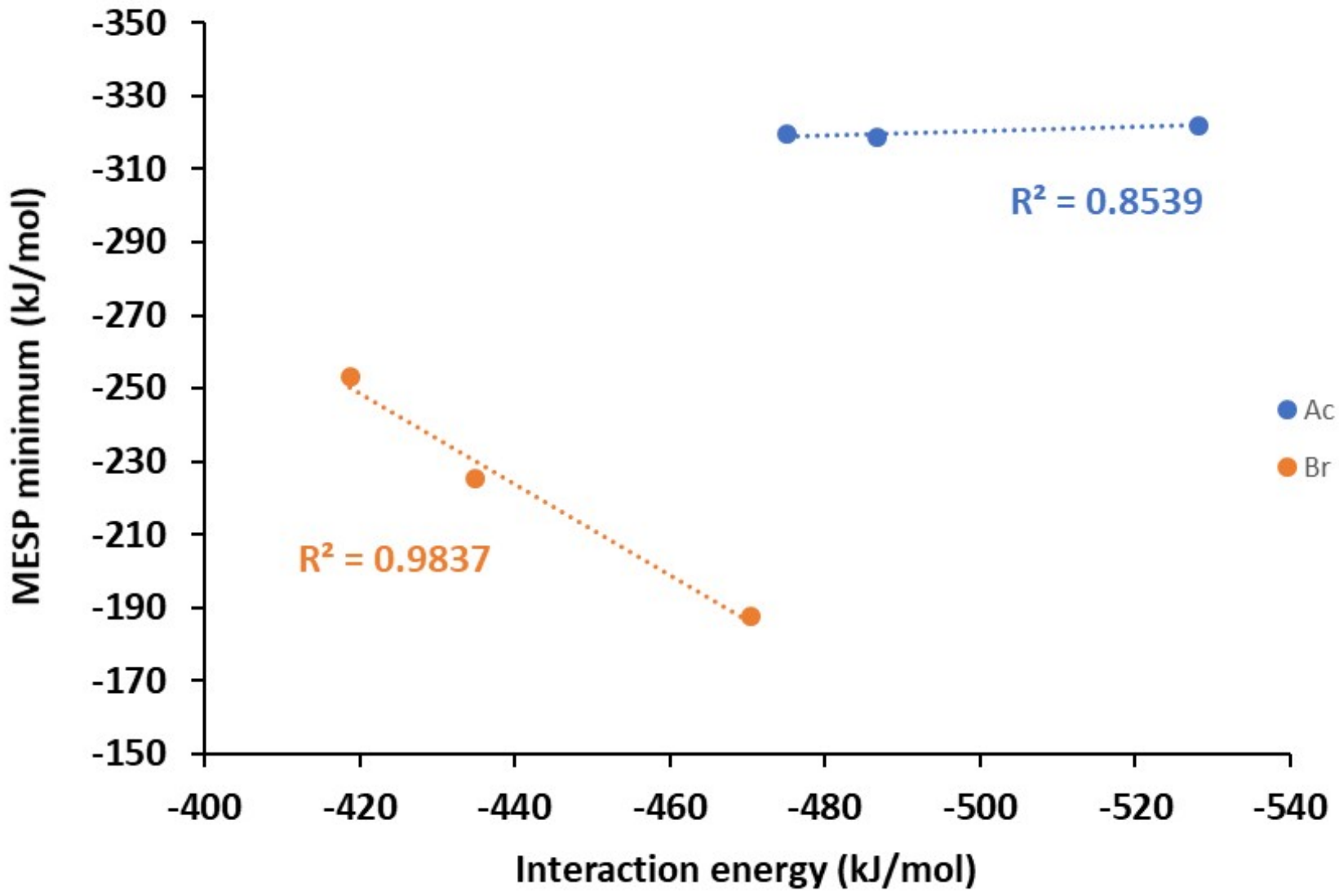


**Figure S5**. Correlation between the interaction energy, ΔE$_1$(KJ/mol) of IL…$CO_2$ complexes with the $V_{min}$ values of corresponding ILs (kJ/mol) at the M06-2X/6-311++G** level.

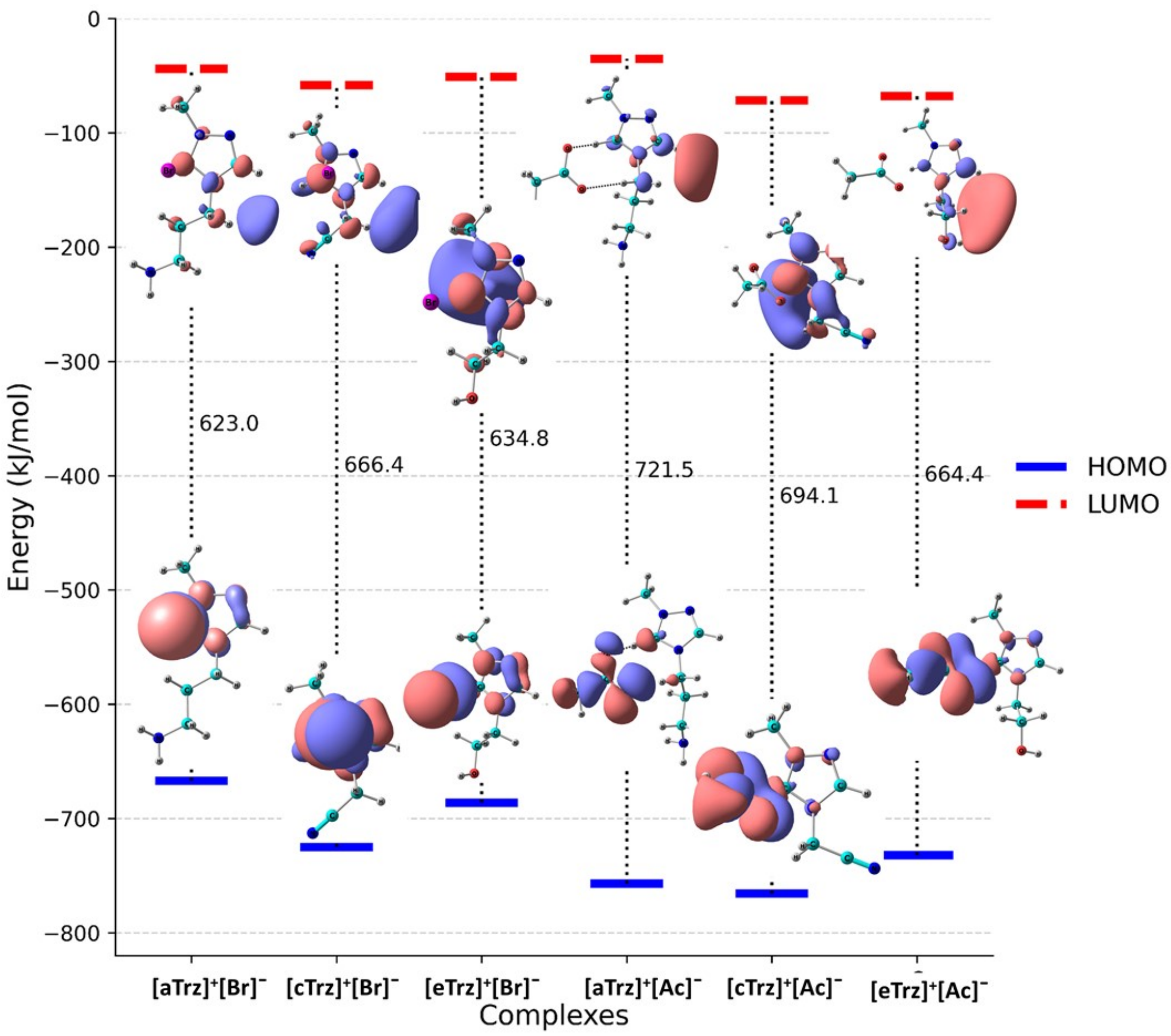


**Figure S6.** HOMO, LUMO (isosurface = 0.03 au) and, and gaps of ILs. Red and blue colour distributions represent positive and negative phases in the molecular orbital wave function, respectively. The numbers on the arrows represent the values of the HOMO-LUMO gap in kJ/mol.

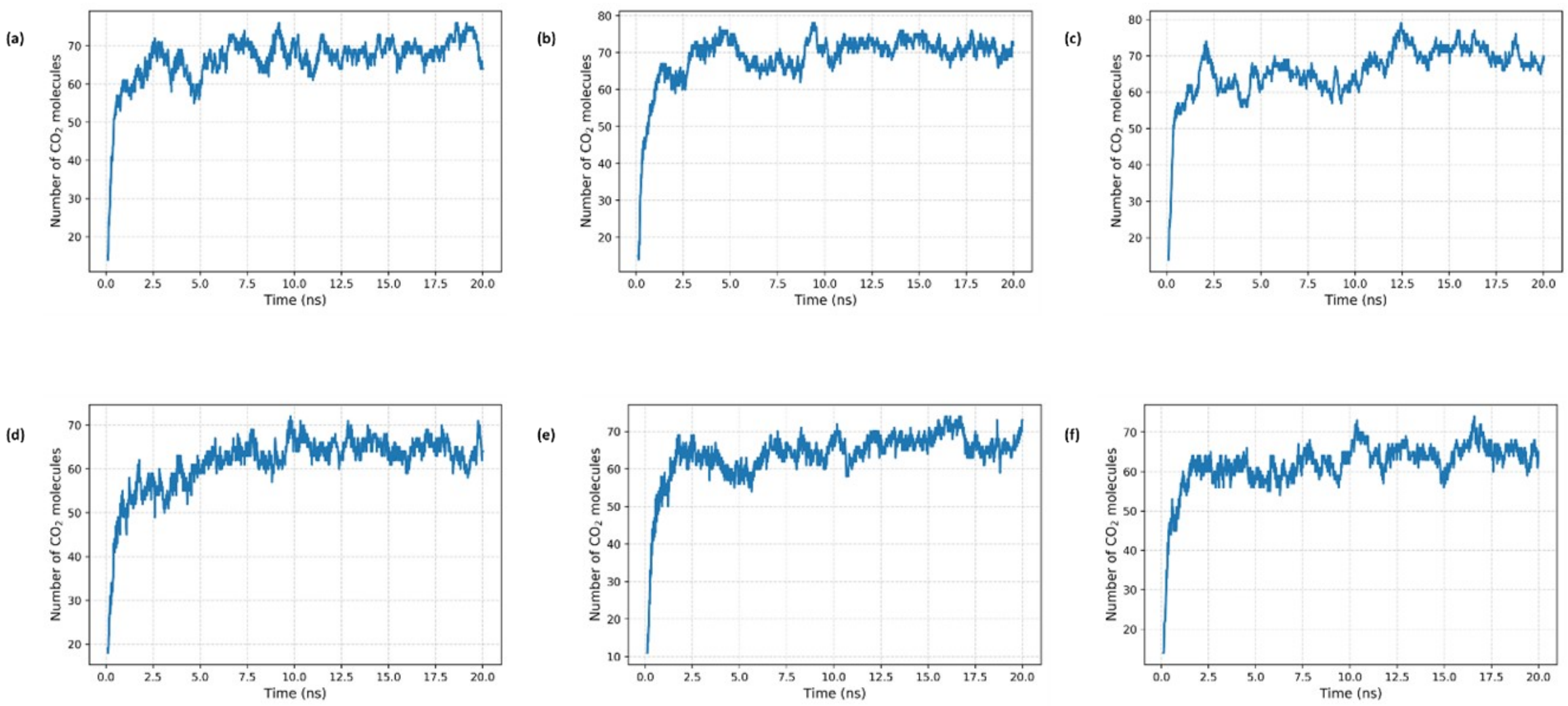


**Figure S7.** Evolution of number of $CO_2$ molecules absorbed by the copolymer with simulation time (a) CP1, (b) CP2, (c) CP3, (d) CP4, (e) CP5, and (f) CP6.

**Table S2**. Number of $CO_2$ molecules in the beginning of MD simulation (N), corresponding pressure (P, calculated using ideal gas equation), number of $CO_2$ molecules adsorbed on the polymer ($N_{adss}$) and number of $CO_2$ molecules in the gas phase after adsorbed by the polymer ($N_{gas}$) and adsorbed on the polymer, concentration of adsorbed $CO_2$ per unit volume ($C_{ads}$) and concentration of $CO_2$ in the gas phase per unit volume ($C_{gas}$) and the free energy of absorption ($\Delta G_{abs}$) for CP1 at various $CO_2$ gas pressures.

| N | P (bar) | $N_{ads}$ | $N_{gas}$ | $C_{gas}$ (mol/m³) | $C_{ads}$ (mol/m³) | $C_{ads}/C_{gas}$ | $\Delta G_{abs}$ (kJ/mol) |
|---|---|---|---|---|---|---|---|
| 13 | 0.15 | 11 | 2 | 0.9 | 165.2 | 176.0 | -12.9 |
| 43 | 0.5 | 39 | 4 | 1.9 | 585.6 | 312.0 | -14.3 |
| 87 | 1 | 70 | 17 | 8.0 | 1050.9 | 131.7 | -12.2 |
| 100 | 1.17 | 89 | 12 | 5.6 | 1336.4 | 237.3 | -13.6 |
| 150 | 1.76 | 144 | 7 | 3.3 | 2162.2 | 658.3 | -16.2 |
| 250 | 2.93 | 167 | 84 | 39.4 | 2507.6 | 63.6 | -10.4 |
| 350 | 4.1 | 217 | 134 | 62.9 | 3258.3 | 51.8 | -9.8 |
| 450 | 5.27 | 264 | 187 | 87.7 | 3964.1 | 45.2 | -9.5 |
| 500 | 5.85 | 289 | 211 | 99.0 | 4339.4 | 43.8 | -9.4 |
| 700 | 8.19 | 358 | 342 | 160.5 | 5375.5 | 33.5 | -8.8 |
| 2000 | 23.4 | 484 | 1516 | 711.4 | 7267.4 | 10.2 | -5.8 |

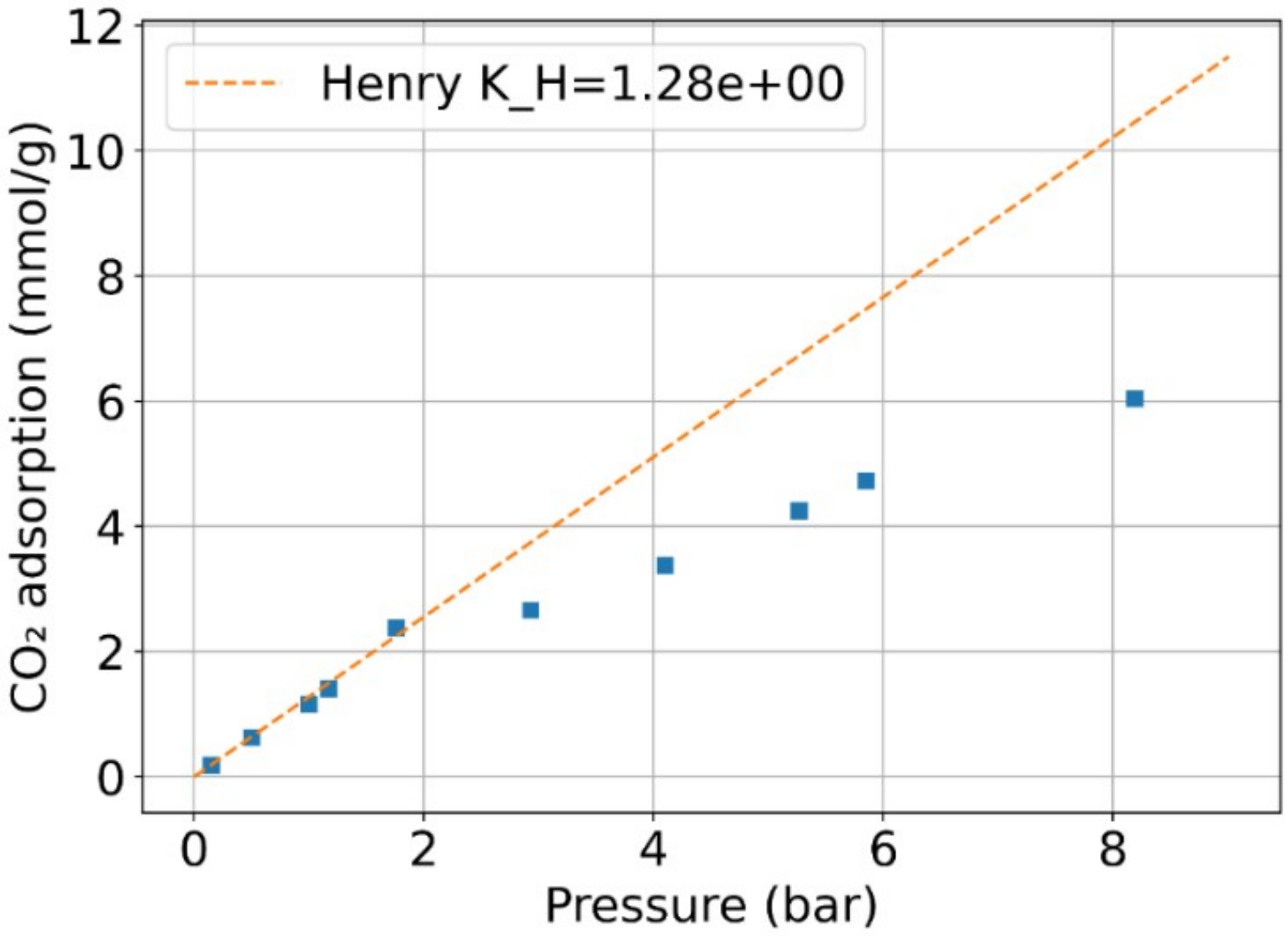


**Figure S8**. Adsorption isotherm of $CO_2$ in [aTrz]+[Br]⁻ based copolymer.

The simulated $CO_2$ adsorption isotherms exhibit Type I-like behaviour, characterized by an approximately linear Henry-law regime at low pressure followed by a progressively reduced adsorption slope at higher pressures. The absence of a distinct plateau within the investigated pressure range suggests that the adsorption sites are not fully saturated under the simulated conditions.

### Free energy calculation

In the "copolymer-gas interfacial system" simulations, the cell dimension is 4.8×4.8 × 158.4 $nm^3$.

Total volume of the simulation box = 4.8×4.8×158.4 = 3649.536 $nm^3$

Gas phase volume ($V_{gas}$) = $L_x$×$L_y$×gas-length = [4.8×4.8×(158.4-4.8)] $nm^3$

= [4.8×4.8×153.6] = 3538.944 $nm^3$ = $3.5389\times10^{-24}$ $m^3$

Total volume of the simulation box = 4.8×4.8×158.4 = 3649.536 $nm^3$

Volume of the adsorbed phase (copolymer) ($V_{ads}$) = 110.592 $nm^3$ = $1.10592\times10^{-25}$ $m^3$

For each phase $C = N/(N_A \cdot V)$, where $N_A = 6.022\text{x}10^{23}$

And, $\Delta G_{ads} = -RT\ln(C_{ads}/C_{gas})$, at T = 300 K

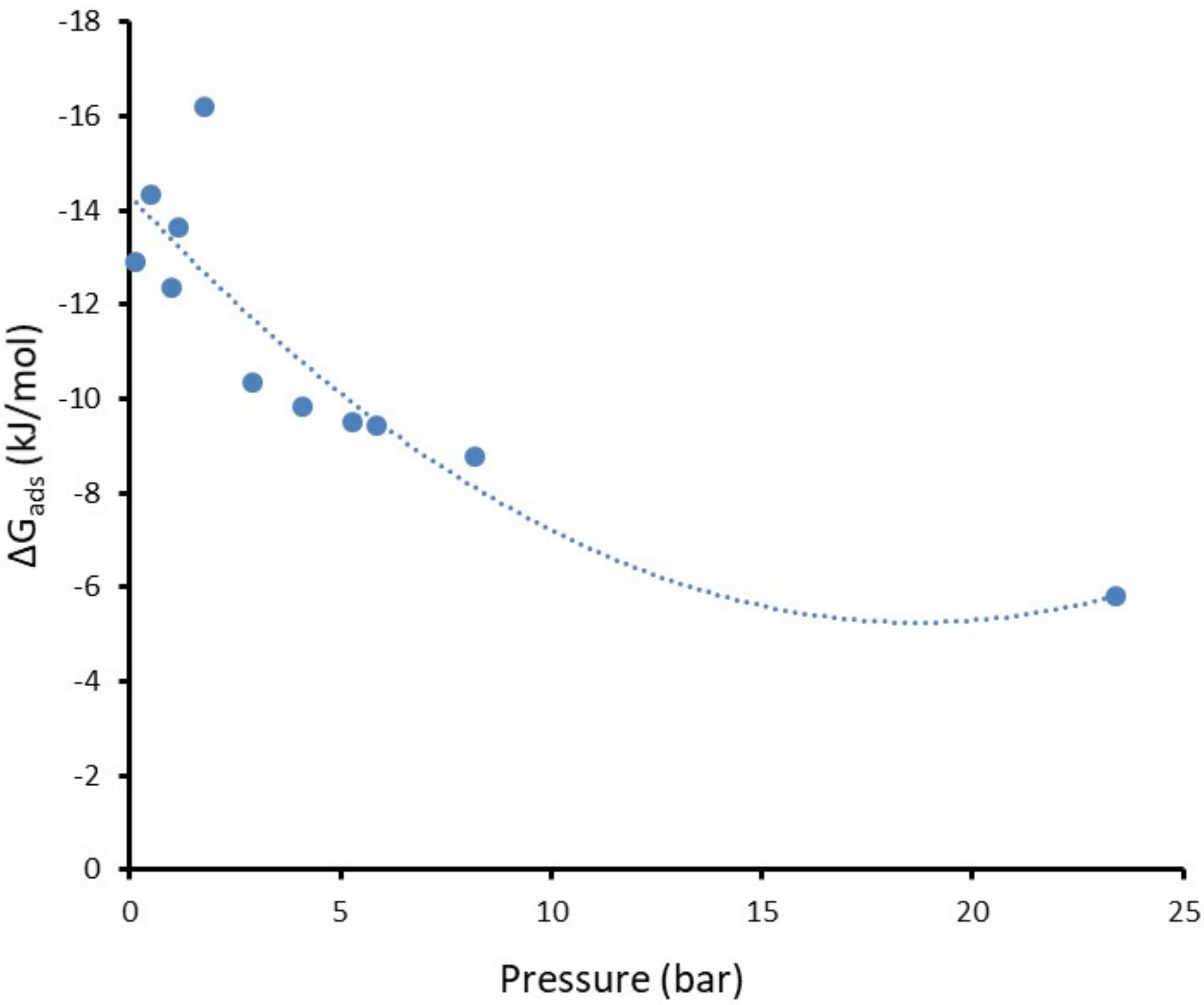


**Figure S9.** $\Delta G_{abs}$ of $CO_2$ on CP1 at various $CO_2$ loading pressures as given in the Table S2.

**Table S3**. Number of $CO_2$ molecules adsorbed on the polymer ($N_{adss}$) and number of $CO_2$ molecules in the gas phase after adsorbed by the polymer ($N_{gas}$) and adsorbed on the polymer, concentration of adsorbed $CO_2$ per unit volume ($C_{ads}$) and concentration of $CO_2$ in the gas phase per unit volume ($C_{gas}$) and the free energy of absorption ($\Delta G_{abs}$) for the six copolymers.

| System | $N_{adss}$ | $N_{gas}$ | $C_{gas}$ (mol/m³) | $C_{ads}$ (mol/m³) | $C_{ads}/C_{gas}$ | $\Delta G_{abs}$ (kJ/mol) |
|---|---|---|---|---|---|---|
| CP1 | 70 | 17 | 8.0 | 1050.9 | 131.7 | -12.2 |
| CP2 | 73 | 14 | 6.6 | 1095.8 | 166.8 | -12.8 |
| CP3 | 70 | 17 | 8.0 | 1050.9 | 131.7 | -12.2 |
| CP4 | 65 | 22 | 10.3 | 976.5 | 94.5 | -11.4 |
| CP5 | 67 | 20 | 9.4 | 1006.6 | 107.2 | -11.7 |
| CP6 | 65 | 22 | 10.3 | 976.5 | 94.5 | -11.4 |

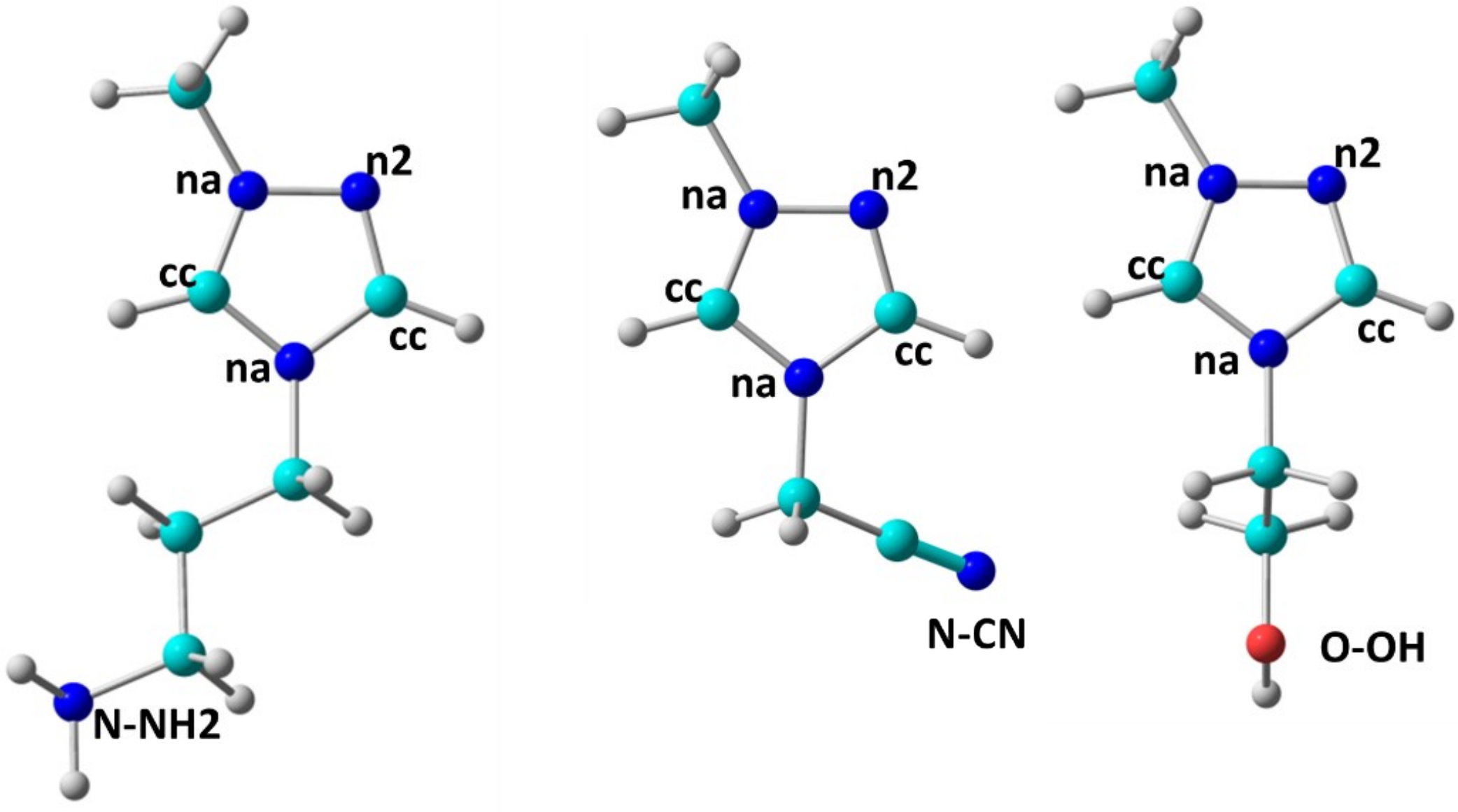


[aTrz]$^+$ [cTrz]$^+$ [eTrz]$^+$

**Figure S10**. The atomic centres on the triazolium parts of the copolymersused in the RDF calculations.

**Table S4**. $CO_2$ self-diffusion coefficients calculated from the mean-square displacement of $CO_2$ molecules in the bulk copolymer– $CO_2$ simulations for the six copolymers.

| System | Diffusion coefficient, $D_{CO2}$ ($\times 10^{-11}$ m²/s) |
|---|---|
| CP1 | 1.87 ± 1.14 |
| CP2 | 1.08 ± 0.39 |
| CP3 | 0.6546 ± 0.99 |
| CP4 | 1.00 ± 0.10 |
| CP5 | 1.57 ± 0.64 |
| CP6 | 0.9978 ± 0.72 |

**Table S5**. Cartesian coordinates of the optimized geometries of 1,2,4-triazolium cations and DVB.

| **[aTrz]$^+$** | | | | **[cTrz]$^+$** | | | |
|---|---|---|---|---|---|---|---|
| 7 | -2.342852893 | -0.449928193 | -0.004956274 | 7 | 1.775257376 | -0.012359464 | -0.042590831 |
| 6 | -1.030748908 | -0.591101042 | -0.062565155 | 6 | 0.867507252 | 0.941657685 | -0.054513723 |
| 7 | -2.693063984 | 0.848884976 | 0.074157841 | 7 | 1.215069282 | -1.223594327 | 0.152158039 |
| 6 | -1.564667056 | 1.494623039 | 0.062980030 | 6 | -0.058349729 | -0.992952953 | 0.262290937 |
| 7 | -0.501746817 | 0.635767835 | -0.020726285 | 7 | -0.319488647 | 0.349208829 | 0.138352863 |
| 1 | -0.491622069 | -1.522412253 | -0.128737183 | 1 | 1.044682675 | 1.997101179 | -0.195678105 |
| 1 | -1.464699124 | 2.567788150 | 0.113709078 | 1 | -0.822417117 | -1.741490047 | 0.413772158 |
| 6 | 0.931785354 | 1.012408154 | -0.072173956 | 6 | -1.626704622 | 1.026734041 | 0.243927957 |
| 1 | 1.084210098 | 1.554716937 | -1.007326994 | 1 | -1.634057540 | 1.878828476 | -0.437058073 |
| 1 | 1.103129243 | 1.695891372 | 0.761191839 | 1 | -1.771612932 | 1.377502355 | 1.267477409 |
| 6 | -3.375016293 | -1.488024199 | -0.015734027 | 6 | 3.226343415 | 0.100748469 | -0.221177040 |
| 1 | -3.951806265 | -1.409556209 | 0.903290218 | 1 | 3.476612503 | 1.150592738 | -0.355363161 |

| | | | |
|---|---|---|---|
| 1 | -4.016515117 | -1.326960107 | -0.879478300 |
| 1 | -2.887066185 | -2.458037318 | -0.078510853 |
| 6 | 1.843814935 | -0.198843114 | 0.012385923 |
| 1 | 1.667702100 | -0.881402918 | -0.823706193 |
| 1 | 1.656867196 | -0.744077188 | 0.944136881 |
| 6 | 3.317670412 | 0.213786021 | -0.036020037 |
| 1 | 3.512120297 | 0.721543233 | -0.984100876 |
| 1 | 3.523541529 | 0.936270132 | 0.766105779 |
| 7 | 4.140203420 | -0.984550147 | 0.016226162 |
| 1 | 4.236155426 | -1.331164949 | 0.964047175 |
| 1 | 5.073180481 | -0.810918096 | -0.336768930 |

| | | | |
|---|---|---|---|
| 1 | 3.710222563 | -0.299372503 | 0.667240122 |
| 1 | 3.507548205 | -0.476894537 | -1.099185815 |
| 6 | -2.689788906 | 0.092221307 | -0.115942206 |
| 7 | -3.502982993 | -0.669929930 | -0.383451889 |

**[eTrz]⁺**

| | | | |
|---|---|---|---|
| 7 | 1.963153929 | -0.179366216 | 0.052247257 |
| 6 | 0.843527056 | -0.788555065 | -0.289892822 |
| 7 | 1.773299420 | 1.147235109 | 0.198408130 |
| 6 | 0.512762171 | 1.331754982 | -0.062029099 |
| 7 | -0.105006758 | 0.150394820 | -0.373954745 |
| 1 | 0.716332954 | -1.843982155 | -0.475940959 |
| 1 | 0.008744125 | 2.286051007 | -0.047239653 |
| 6 | -1.530073152 | -0.049608250 | -0.685405719 |
| 1 | -1.626745898 | -0.972346261 | -1.256873755 |
| 1 | -1.856635841 | 0.779109780 | -1.313269762 |
| 6 | 3.293356305 | -0.755782060 | 0.261053191 |
| 1 | 3.231645242 | -1.830265880 | 0.104374921 |
| 1 | 3.979435138 | -0.304001217 | -0.452230114 |
| 1 | 3.606558327 | -0.534993965 | 1.279162172 |
| 6 | -2.370188073 | -0.121866875 | 0.586098200 |
| 1 | -2.271046187 | 0.813243830 | 1.151557429 |
| 1 | -2.017740150 | -0.948723260 | 1.215774146 |
| 8 | -3.673898249 | -0.325934026 | 0.118537291 |
| 1 | -4.295792114 | -0.360125227 | 0.850545005 |

**DVB**

| | | | |
|---|---|---|---|
| 6 | 0.215232000 | -0.040728000 | 1.414446000 |
| 6 | -0.983100000 | -0.128392000 | 0.691258000 |
| 6 | 1.409482000 | 0.022563000 | 0.694561000 |
| 1 | -1.924991000 | -0.218911000 | 1.219353000 |
| 1 | 2.350339000 | 0.077676000 | 1.231954000 |
| 6 | -0.983100000 | -0.128392000 | -0.691258000 |
| 6 | 1.409482000 | 0.022563000 | -0.694561000 |
| 1 | -1.924991000 | -0.218911000 | -1.219353000 |
| 1 | 2.350339000 | 0.077676000 | -1.231954000 |
| 6 | 0.215232000 | -0.040728000 | -1.414446000 |
| 6 | 0.259014000 | -0.022363000 | -2.886144000 |
| 1 | 1.250118000 | -0.143564000 | -3.316517000 |
| 6 | -0.775989000 | 0.143469000 | -3.708377000 |
| 1 | -1.786968000 | 0.294542000 | -3.347530000 |
| 1 | -0.636330000 | 0.142961000 | -4.781977000 |
| 6 | 0.259014000 | -0.022363000 | 2.886144000 |
| 1 | 1.250118000 | -0.143564000 | 3.316517000 |
| 6 | -0.775989000 | 0.143469000 | 3.708377000 |
| 1 | -1.786968000 | 0.294542000 | 3.347530000 |
| 1 | -0.636330000 | 0.142961000 | 4.781977000 |

**Table S6**. Cartesian coordinates of the oligomers representing the copolymers.

Oligomer 1

| | | | |
|---|---|---|---|
| 6 | 6.239351000 | 1.026045000 | 0.248293000 |
| 6 | 6.297190000 | 1.451706000 | -1.089926000 |
| 6 | 6.418118000 | 2.001927000 | 1.240723000 |
| 1 | 6.140545000 | 0.724130000 | -1.882735000 |
| 1 | 6.374600000 | 1.725234000 | 2.289860000 |
| 6 | 6.568250000 | 2.774169000 | -1.426982000 |
| 6 | 6.677746000 | 3.330258000 | 0.909717000 |
| 1 | 6.598399000 | 3.061035000 | -2.474604000 |
| 1 | 6.817534000 | 4.060447000 | 1.704029000 |
| 6 | 6.791616000 | 3.740220000 | -0.429534000 |
| 6 | 7.171329000 | 5.131934000 | -0.727211000 |
| 1 | 7.052906000 | 5.828530000 | 0.103561000 |
| 6 | 7.665201000 | 5.603905000 | -1.882918000 |
| 1 | 7.812009000 | 4.973769000 | -2.757095000 |
| 1 | 7.901265000 | 6.656994000 | -2.002138000 |
| 6 | 6.045265000 | -0.451031000 | 0.572891000 |
| 1 | 6.138345000 | -0.984564000 | -0.381841000 |
| 6 | 4.606221000 | -0.817009000 | 1.051484000 |
| 1 | 3.941482000 | -0.632393000 | 0.198098000 |
| 1 | 4.585797000 | -1.903462000 | 1.216143000 |
| 6 | 8.659029000 | -1.872915000 | -0.438384000 |
| 6 | 8.566822000 | -3.268042000 | -0.297193000 |
| 6 | 8.814342000 | -1.363832000 | -1.732955000 |
| 1 | 8.445587000 | -3.704911000 | 0.691272000 |
| 1 | 8.872078000 | -0.286958000 | -1.879265000 |
| 6 | 8.637828000 | -4.113126000 | -1.397114000 |
| 6 | 8.883386000 | -2.209508000 | -2.840230000 |
| 1 | 8.564745000 | -5.185436000 | -1.242770000 |

Oligomer 2

| | | | |
|---|---|---|---|
| 6 | 6.433545000 | 1.386475000 | -0.066090000 |
| 6 | 6.528006000 | 1.593403000 | -1.453595000 |
| 6 | 6.529828000 | 2.519317000 | 0.758040000 |
| 1 | 6.434199000 | 0.740641000 | -2.121560000 |
| 1 | 6.458224000 | 2.414520000 | 1.836201000 |
| 6 | 6.752377000 | 2.856019000 | -1.995115000 |
| 6 | 6.734416000 | 3.789801000 | 0.221750000 |
| 1 | 6.811844000 | 2.969381000 | -3.074116000 |
| 1 | 6.800697000 | 4.646462000 | 0.889556000 |
| 6 | 6.883927000 | 3.984231000 | -1.164078000 |
| 6 | 7.218913000 | 5.326491000 | -1.673106000 |
| 1 | 7.029869000 | 6.143848000 | -0.976628000 |
| 6 | 7.752195000 | 5.624091000 | -2.869144000 |
| 1 | 7.964248000 | 4.866669000 | -3.620516000 |
| 1 | 7.944373000 | 6.653921000 | -3.154661000 |
| 6 | 6.273463000 | -0.025020000 | 0.488592000 |
| 1 | 6.378255000 | -0.700020000 | -0.370245000 |
| 6 | 4.840590000 | -0.344538000 | 1.017563000 |
| 1 | 4.171405000 | -0.291519000 | 0.149404000 |
| 1 | 4.843730000 | -1.398688000 | 1.327464000 |
| 6 | 8.814026000 | -1.716849000 | -0.243267000 |
| 6 | 8.700140000 | -3.042371000 | 0.208845000 |
| 6 | 8.919295000 | -1.512845000 | -1.623878000 |
| 1 | 8.615048000 | -3.245429000 | 1.273847000 |
| 1 | 8.997398000 | -0.497828000 | -2.009417000 |
| 6 | 8.704135000 | -4.111949000 | -0.677153000 |
| 6 | 8.920417000 | -2.584109000 | -2.517049000 |
| 1 | 8.616823000 | -5.120684000 | -0.285016000 |
| 1 | 9.003799000 | -2.392795000 | -3.584603000 |

| | | | |
|---|---|---|---|
| 1 | 9.002584000 | -1.783617000 | -3.834144000 |
| 6 | 8.802131000 | -3.603420000 | -2.699371000 |
| 6 | 8.887562000 | -4.453324000 | -3.897625000 |
| 1 | 8.958177000 | -3.905054000 | -4.837058000 |
| 6 | 8.893247000 | -5.791222000 | -3.946668000 |
| 1 | 8.835762000 | -6.416465000 | -3.059798000 |
| 1 | 8.961903000 | -6.313313000 | -4.895803000 |
| 6 | 8.579331000 | -0.942022000 | 0.763885000 |
| 1 | 8.705343000 | 0.083079000 | 0.389297000 |
| 6 | 7.193614000 | -0.995110000 | 1.459795000 |
| 1 | 6.963106000 | -2.030413000 | 1.742517000 |
| 1 | 7.258319000 | -0.429026000 | 2.395135000 |
| 6 | 2.475190000 | -0.428299000 | 2.336461000 |
| 6 | 2.007711000 | -1.723990000 | 2.598448000 |
| 6 | 1.521115000 | 0.582119000 | 2.158724000 |
| 1 | 2.714421000 | -2.536819000 | 2.746821000 |
| 1 | 1.851489000 | 1.598626000 | 1.955969000 |
| 6 | 0.642597000 | -1.991365000 | 2.697074000 |
| 6 | 0.155498000 | 0.316019000 | 2.258034000 |
| 1 | 0.312801000 | -3.003863000 | 2.921662000 |
| 1 | -0.557236000 | 1.128538000 | 2.131033000 |
| 6 | -0.308161000 | -0.975724000 | 2.537514000 |
| 6 | -1.790476000 | -1.260166000 | 2.668913000 |
| 1 | -2.266763000 | -0.456543000 | 3.246496000 |
| 6 | -2.517551000 | -1.384538000 | 1.315004000 |
| 1 | -2.063858000 | -2.198494000 | 0.731596000 |
| 1 | -2.369279000 | -0.466048000 | 0.739553000 |
| 6 | 3.968026000 | -0.111961000 | 2.276382000 |
| 1 | 4.052097000 | 0.970709000 | 2.119127000 |
| 6 | 4.634335000 | -0.446108000 | 3.626541000 |
| 1 | 4.670222000 | -1.527919000 | 3.800199000 |
| 1 | 5.660289000 | -0.070295000 | 3.683502000 |
| 6 | 11.856087000 | -2.182946000 | 0.741095000 |
| 6 | 12.121073000 | -3.237165000 | 1.631010000 |
| 6 | 12.314299000 | -2.310537000 | -0.573511000 |
| 1 | 11.780624000 | -3.177338000 | 2.662881000 |
| 1 | 12.109810000 | -1.521631000 | -1.293831000 |
| 6 | 12.811846000 | -4.371342000 | 1.221758000 |
| 6 | 13.003654000 | -3.448709000 | -0.987397000 |
| 1 | 12.994184000 | -5.164598000 | 1.940377000 |
| 1 | 13.337686000 | -3.525119000 | -2.019272000 |
| 6 | 13.272250000 | -4.504385000 | -0.101558000 |
| 6 | 14.007468000 | -5.682127000 | -0.589499000 |
| 1 | 14.272865000 | -5.644025000 | -1.645651000 |
| 6 | 14.373315000 | -6.759971000 | 0.115135000 |
| 1 | 14.153864000 | -6.879869000 | 1.172676000 |
| 1 | 14.918077000 | -7.572944000 | -0.354187000 |
| 6 | 11.143623000 | -0.917163000 | 1.206624000 |
| 1 | 11.030624000 | -0.273942000 | 0.322008000 |
| 6 | 9.720938000 | -1.170132000 | 1.786431000 |
| 1 | 9.532702000 | -0.499571000 | 2.635554000 |
| 1 | 9.666679000 | -2.185151000 | 2.195865000 |
| 6 | 12.369174000 | 2.650210000 | 0.661003000 |
| 7 | 11.461780000 | 2.178943000 | 1.507080000 |
| 7 | 10.228501000 | 2.684059000 | 1.228869000 |
| 6 | 11.638422000 | 1.232776000 | 2.634988000 |
| 7 | 11.733413000 | 3.477416000 | -0.188226000 |
| 1 | 13.423409000 | 2.415810000 | 0.653504000 |
| 6 | 10.415939000 | 3.470163000 | 0.197802000 |
| 1 | 9.639172000 | 4.045415000 | -0.291897000 |
| 6 | 12.346746000 | 4.279406000 | -1.275706000 |
| 1 | 12.943183000 | 3.595341000 | -1.886694000 |
| 1 | 11.515881000 | 4.635151000 | -1.890314000 |
| 1 | 12.372857000 | 1.675224000 | 3.314469000 |
| 1 | 10.669199000 | 1.233997000 | 3.133556000 |
| 1 | 4.071322000 | 0.002565000 | 4.451665000 |
| 6 | -9.159040000 | 2.285086000 | 0.517137000 |
| 6 | -9.108157000 | 3.661021000 | 0.239869000 |
| 6 | -8.696503000 | 1.868627000 | 1.775012000 |
| 1 | -9.464918000 | 4.020144000 | -0.723026000 |
| 1 | -8.723379000 | 0.815951000 | 2.045330000 |
| 6 | 8.818277000 | -3.908971000 | -2.065905000 |
| 6 | 8.831694000 | -5.005643000 | -3.046950000 |
| 1 | 8.868212000 | -4.682052000 | -4.087096000 |
| 6 | 8.811158000 | -6.320479000 | -2.795799000 |
| 1 | 8.784965000 | -6.730966000 | -1.790038000 |
| 1 | 8.825992000 | -7.042353000 | -3.606209000 |
| 6 | 8.798680000 | -0.539562000 | 0.722286000 |
| 1 | 8.930236000 | 0.371405000 | 0.122483000 |
| 6 | 7.433392000 | -0.398907000 | 1.448184000 |
| 1 | 7.188013000 | -1.342083000 | 1.952467000 |
| 1 | 7.537155000 | 0.354623000 | 2.236587000 |
| 6 | 2.709896000 | 0.144142000 | 2.267634000 |
| 6 | 2.296743000 | -1.121605000 | 2.707060000 |
| 6 | 1.715109000 | 1.086508000 | 1.976004000 |
| 1 | 3.036003000 | -1.881230000 | 2.948878000 |
| 1 | 2.002833000 | 2.078967000 | 1.635658000 |
| 6 | 0.944539000 | -1.423805000 | 2.864183000 |
| 6 | 0.362153000 | 0.785865000 | 2.134041000 |
| 1 | 0.657641000 | -2.409313000 | 3.225681000 |
| 1 | -0.383019000 | 1.548004000 | 1.914581000 |
| 6 | -0.047002000 | -0.474067000 | 2.588757000 |
| 6 | -1.515294000 | -0.795325000 | 2.780557000 |
| 1 | -2.018156000 | 0.058236000 | 3.254560000 |
| 6 | -2.249127000 | -1.126165000 | 1.465657000 |
| 1 | -1.761323000 | -1.984645000 | 0.982428000 |
| 1 | -2.154080000 | -0.281736000 | 0.776286000 |
| 6 | 4.188746000 | 0.504055000 | 2.139472000 |
| 1 | 4.227261000 | 1.555950000 | 1.829383000 |
| 6 | 4.882680000 | 0.390126000 | 3.512010000 |
| 1 | 4.953899000 | -0.653259000 | 3.840394000 |
| 1 | 5.897653000 | 0.798669000 | 3.495726000 |
| 6 | 12.018233000 | -1.785611000 | 0.747313000 |
| 6 | 12.263210000 | -2.741567000 | 1.746896000 |
| 6 | 12.442156000 | -2.079584000 | -0.551897000 |
| 1 | 11.951543000 | -2.550704000 | 2.771945000 |
| 1 | 12.258139000 | -1.367075000 | -1.352941000 |
| 6 | 12.898787000 | -3.942706000 | 1.457048000 |
| 6 | 13.076706000 | -3.284728000 | -0.845921000 |
| 1 | 13.066924000 | -4.655952000 | 2.258198000 |
| 1 | 13.386168000 | -3.490035000 | -1.867730000 |
| 6 | 13.322957000 | -4.243976000 | 0.149355000 |
| 6 | 14.000462000 | -5.498667000 | -0.214293000 |
| 1 | 14.242664000 | -5.591883000 | -1.272513000 |
| 6 | 14.340800000 | -6.503087000 | 0.602635000 |
| 1 | 14.141913000 | -6.492421000 | 1.670936000 |
| 1 | 14.843122000 | -7.385696000 | 0.219771000 |
| 6 | 11.373942000 | -0.442623000 | 1.079914000 |
| 1 | 11.246369000 | 0.091490000 | 0.126953000 |
| 6 | 9.969602000 | -0.555681000 | 1.739101000 |
| 1 | 9.806176000 | 0.285745000 | 2.423968000 |
| 1 | 9.923618000 | -1.458199000 | 2.359216000 |
| 6 | 12.327172000 | 3.288773000 | 0.251670000 |
| 7 | 11.596167000 | 2.640905000 | 1.146339000 |
| 7 | 10.265373000 | 2.785066000 | 0.891189000 |
| 6 | 12.030235000 | 1.813784000 | 2.298818000 |
| 7 | 11.465661000 | 3.872180000 | -0.608147000 |
| 1 | 13.405035000 | 3.364323000 | 0.224256000 |
| 6 | 10.204962000 | 3.534241000 | -0.177446000 |
| 1 | 9.288262000 | 3.854802000 | -0.656592000 |
| 6 | 11.786793000 | 4.699199000 | -1.792537000 |
| 1 | 11.994210000 | 4.043627000 | -2.645174000 |
| 1 | 10.896007000 | 5.293032000 | -2.023735000 |
| 1 | 12.883550000 | 2.324882000 | 2.752754000 |
| 1 | 11.191943000 | 1.864213000 | 2.993823000 |
| 1 | 4.316639000 | 0.937772000 | 4.272723000 |
| 6 | -9.236998000 | 1.830745000 | 0.453564000 |
| 6 | -9.354638000 | 3.181412000 | 0.086695000 |
| 6 | -8.711807000 | 1.557215000 | 1.725894000 |
| 1 | -9.763653000 | 3.430635000 | -0.890019000 |
| 1 | -8.609452000 | 0.529263000 | 2.065078000 |
| 6 | -8.967919000 | 4.209489000 | 0.939807000 |

| | | | | | | | |
|---|---|---|---|---|---|---|---|
| 6 | -8.619023000 | 4.577919000 | 1.164254000 | 6 | -8.326329000 | 2.585392000 | 2.585951000 |
| 6 | -8.209360000 | 2.785277000 | 2.706401000 | 1 | -9.085603000 | 5.238131000 | 0.612243000 |
| 1 | -8.607001000 | 5.632390000 | 0.904790000 | 1 | -7.937353000 | 2.339737000 | 3.572116000 |
| 1 | -7.872295000 | 2.431230000 | 3.678619000 | 6 | -8.438517000 | 3.935523000 | 2.213378000 |
| 6 | -8.151563000 | 4.160272000 | 2.422852000 | 6 | -8.008860000 | 4.979906000 | 3.157169000 |
| 6 | -7.617729000 | 5.081844000 | 3.438634000 | 1 | -7.699590000 | 4.607882000 | 4.134123000 |
| 1 | -7.379325000 | 4.613675000 | 4.393894000 | 6 | -7.960239000 | 6.300373000 | 2.941170000 |
| 6 | -7.400196000 | 6.396696000 | 3.309531000 | 1 | -8.246924000 | 6.756284000 | 1.997368000 |
| 1 | -7.606005000 | 6.942796000 | 2.392869000 | 1 | -7.630772000 | 6.980615000 | 3.720240000 |
| 1 | -7.006975000 | 6.980254000 | 4.136044000 | 6 | -9.694215000 | 0.737580000 | -0.506292000 |
| 6 | -9.720752000 | 1.318059000 | -0.519376000 | 1 | -9.821447000 | 1.220039000 | -1.484636000 |
| 1 | -9.780327000 | 1.878267000 | -1.462156000 | 6 | -11.120238000 | 0.196700000 | -0.179638000 |
| 6 | -11.202437000 | 0.918192000 | -0.241942000 | 1 | -11.802763000 | 1.048775000 | -0.279506000 |
| 1 | -11.786357000 | 1.844650000 | -0.295910000 | 1 | -11.398835000 | -0.504702000 | -0.978991000 |
| 1 | -11.541518000 | 0.301539000 | -1.086534000 | 6 | -7.777257000 | 0.258572000 | -3.048779000 |
| 6 | -7.854345000 | 0.806990000 | -3.086790000 | 6 | -8.190230000 | -0.844386000 | -3.815752000 |
| 6 | -8.402546000 | -0.182584000 | -3.920916000 | 6 | -7.688501000 | 1.497781000 | -3.692597000 |
| 6 | -7.603393000 | 2.062561000 | -3.651431000 | 1 | -8.275866000 | -1.825613000 | -3.352388000 |
| 1 | -8.616818000 | -1.171491000 | -3.519992000 | 1 | -7.370125000 | 2.371928000 | -3.129074000 |
| 1 | -7.178034000 | 2.850917000 | -3.034532000 | 6 | -8.500098000 | -0.714855000 | -5.164213000 |
| 6 | -8.687171000 | 0.071345000 | -5.257267000 | 6 | -8.002105000 | 1.631463000 | -5.044531000 |
| 6 | -7.890493000 | 2.321063000 | -4.991091000 | 1 | -8.816404000 | -1.592236000 | -5.720674000 |
| 1 | -9.112063000 | -0.720617000 | -5.867118000 | 1 | -7.927704000 | 2.608726000 | -5.515530000 |
| 1 | -7.688682000 | 3.308545000 | -5.399375000 | 6 | -8.414686000 | 0.531448000 | -5.812944000 |
| 6 | -8.438752000 | 1.334686000 | -5.826015000 | 6 | -8.734916000 | 0.728373000 | -7.235825000 |
| 6 | -8.725418000 | 1.661379000 | -7.232074000 | 1 | -8.615253000 | 1.752540000 | -7.588097000 |
| 1 | -8.464769000 | 2.679164000 | -7.521255000 | 6 | -9.147861000 | -0.195812000 | -8.111846000 |
| 6 | -9.260825000 | 0.859617000 | -8.160876000 | 1 | -9.297357000 | -1.239159000 | -7.846913000 |
| 1 | -9.555252000 | -0.166960000 | -7.959373000 | 1 | -9.357154000 | 0.069135000 | -9.143319000 |
| 1 | -9.429260000 | 1.214496000 | -9.172646000 | 6 | -7.418093000 | 0.120095000 | -1.576170000 |
| 6 | -7.519582000 | 0.529560000 | -1.627811000 | 1 | -7.121439000 | 1.114478000 | -1.220397000 |
| 1 | -7.118891000 | 1.459279000 | -1.205529000 | 6 | -8.619568000 | -0.353104000 | -0.717351000 |
| 6 | -8.769247000 | 0.132172000 | -0.800189000 | 1 | -9.079779000 | -1.222753000 | -1.205235000 |
| 1 | -9.317620000 | -0.649748000 | -1.341988000 | 1 | -8.249047000 | -0.707483000 | 0.251390000 |
| 1 | -8.444711000 | -0.322649000 | 0.142346000 | 6 | -12.923182000 | -0.547334000 | 1.414132000 |
| 6 | -13.106979000 | 0.269329000 | 1.268756000 | 6 | -13.743485000 | -1.342345000 | 0.596057000 |
| 6 | -13.980888000 | -0.405488000 | 0.399753000 | 6 | -13.537194000 | 0.169939000 | 2.446179000 |
| 6 | -13.673530000 | 1.002708000 | 2.316536000 | 1 | -13.305692000 | -1.912294000 | -0.220635000 |
| 1 | -13.580446000 | -0.984179000 | -0.429805000 | 1 | -12.931996000 | 0.798631000 | 3.095949000 |
| 1 | -13.025804000 | 1.539846000 | 3.006193000 | 6 | -15.115977000 | -1.416970000 | 0.801927000 |
| 6 | -15.358898000 | -0.349825000 | 0.571854000 | 6 | -14.913378000 | 0.096279000 | 2.655611000 |
| 6 | -15.055235000 | 1.059771000 | 2.492151000 | 1 | -15.714926000 | -2.041252000 | 0.145307000 |
| 1 | -15.999667000 | -0.884385000 | -0.123400000 | 1 | -15.361824000 | 0.666117000 | 3.466111000 |
| 1 | -15.465775000 | 1.638384000 | 3.316461000 | 6 | -15.735753000 | -0.697643000 | 1.840662000 |
| 6 | -15.930954000 | 0.386125000 | 1.626135000 | 6 | -17.182902000 | -0.739689000 | 2.104940000 |
| 6 | -17.381490000 | 0.479429000 | 1.856340000 | 1 | -17.506238000 | -0.125174000 | 2.944925000 |
| 1 | -17.663697000 | 1.088889000 | 2.714696000 | 6 | -18.112872000 | -1.434250000 | 1.437826000 |
| 6 | -18.358713000 | -0.090075000 | 1.140026000 | 1 | -17.880675000 | -2.070641000 | 0.588022000 |
| 1 | -18.169143000 | -0.711868000 | 0.269096000 | 1 | -19.158095000 | -1.385185000 | 1.726316000 |
| 1 | -19.400950000 | 0.052463000 | 1.407615000 | 6 | -11.416692000 | -0.458884000 | 1.193941000 |
| 6 | -11.594026000 | 0.216330000 | 1.083984000 | 1 | -11.022840000 | 0.204235000 | 1.973518000 |
| 1 | -11.156069000 | 0.791358000 | 1.908765000 | 6 | -10.767741000 | -1.845938000 | 1.387697000 |
| 6 | -11.089565000 | -1.236844000 | 1.206297000 | 1 | -11.070101000 | -2.539190000 | 0.592794000 |
| 1 | -11.433775000 | -1.849196000 | 0.363536000 | 1 | -9.673361000 | -1.787580000 | 1.386032000 |
| 1 | -9.995577000 | -1.289445000 | 1.234418000 | 6 | -4.576780000 | 0.754884000 | -0.206683000 |
| 6 | -4.667933000 | 0.777926000 | -0.203178000 | 6 | -3.593595000 | 0.887231000 | -1.202806000 |
| 6 | -3.676324000 | 0.924558000 | -1.188919000 | 6 | -4.781493000 | 1.834439000 | 0.659904000 |
| 6 | -4.788651000 | 1.786806000 | 0.758881000 | 1 | -3.412471000 | 0.076914000 | -1.905755000 |
| 1 | -3.558257000 | 0.170039000 | -1.963723000 | 1 | -5.557216000 | 1.779162000 | 1.419584000 |
| 1 | -5.567135000 | 1.723045000 | 1.514826000 | 6 | -2.837704000 | 2.047385000 | -1.317883000 |
| 6 | -2.831662000 | 2.027724000 | -1.202149000 | 6 | -4.026796000 | 3.000820000 | 0.540978000 |
| 6 | -3.945186000 | 2.897037000 | 0.742508000 | 1 | -2.089388000 | 2.111584000 | -2.101803000 |
| 1 | -2.080176000 | 2.105421000 | -1.981878000 | 1 | -4.218798000 | 3.828231000 | 1.219339000 |
| 1 | -4.073710000 | 3.671753000 | 1.494196000 | 6 | -3.036588000 | 3.134874000 | -0.445209000 |
| 6 | -2.945515000 | 3.042636000 | -0.232338000 | 6 | -2.272133000 | 4.389828000 | -0.527817000 |
| 6 | -2.084245000 | 4.235753000 | -0.203650000 | 1 | -2.549388000 | 5.137689000 | 0.214313000 |
| 1 | -2.315110000 | 4.940428000 | 0.594596000 | 6 | -1.304290000 | 4.693722000 | -1.401412000 |
| 6 | -1.079354000 | 4.531373000 | -1.037339000 | 1 | -0.969740000 | 4.010584000 | -2.177545000 |
| 1 | -0.783456000 | 3.887555000 | -1.861300000 | 1 | -0.811779000 | 5.660452000 | -1.370793000 |
| 1 | -0.513257000 | 5.450204000 | -0.921576000 | 6 | -5.417998000 | -0.508975000 | -0.072852000 |
| 6 | -5.604881000 | -0.423893000 | -0.180742000 | 1 | -6.140243000 | -0.331846000 | 0.732916000 |

| | | | |
|---|---|---|---|
| 1 | -6.311328000 | -0.269228000 | 0.643228000 |
| 6 | -6.407210000 | -0.547555000 | -1.500357000 |
| 1 | -6.877267000 | -1.538178000 | -1.580144000 |
| 1 | -5.719110000 | -0.466489000 | -2.349007000 |
| 6 | -5.929001000 | -3.927347000 | -0.467731000 |
| 7 | -5.813409000 | -2.846851000 | 0.291580000 |
| 7 | -6.781622000 | -2.821960000 | 1.246770000 |
| 6 | -4.828675000 | -1.726721000 | 0.186532000 |
| 7 | -6.984450000 | -4.631009000 | -0.017030000 |
| 1 | -5.298231000 | -4.192775000 | -1.302823000 |
| 6 | -7.476237000 | -3.912306000 | 1.042917000 |
| 1 | -8.335058000 | -4.213542000 | 1.625309000 |
| 6 | -7.473629000 | -5.939924000 | -0.514190000 |
| 1 | -7.203516000 | -5.997865000 | -1.572134000 |
| 1 | -8.565219000 | -5.903887000 | -0.456167000 |
| 1 | -4.177979000 | -2.025539000 | -0.643514000 |
| 1 | -11.472464000 | -1.694124000 | 2.125302000 |
| 1 | -1.936535000 | -2.183603000 | 3.244771000 |
| 1 | -7.153459000 | -7.005746000 | 1.349579000 |
| 1 | -5.810038000 | -7.111023000 | 0.211959000 |
| 6 | -7.457057000 | -8.462498000 | -0.202274000 |
| 6 | -6.905765000 | -7.113657000 | 0.287819000 |
| 1 | -8.548452000 | -8.461067000 | -0.093917000 |
| 1 | -7.248366000 | -8.575532000 | -1.282142000 |
| 7 | -6.924717000 | -9.530508000 | 0.637740000 |
| 1 | -5.973045000 | -9.765919000 | 0.362550000 |
| 1 | -7.475186000 | -10.378507000 | 0.525967000 |
| 1 | 13.999510000 | 5.058817000 | -0.100395000 |
| 1 | 12.560790000 | 6.080175000 | -0.105172000 |
| 6 | 13.818930000 | 6.267747000 | -1.864399000 |
| 6 | 13.189417000 | 5.437006000 | -0.734726000 |
| 1 | 14.444894000 | 5.613211000 | -2.483375000 |
| 1 | 13.020338000 | 6.655334000 | -2.523341000 |
| 7 | 14.681649000 | 7.289756000 | -1.280684000 |
| 1 | 14.129187000 | 8.067345000 | -0.923985000 |
| 1 | 15.301693000 | 7.677615000 | -1.987711000 |
| 6 | -4.018315000 | -1.659089000 | 1.506283000 |
| 1 | -4.460428000 | -0.890083000 | 2.149370000 |
| 1 | -4.132663000 | -2.609009000 | 2.042791000 |
| 6 | 12.076399000 | -0.170563000 | 2.196865000 |
| 1 | 12.185853000 | -0.750908000 | 3.121337000 |
| 1 | 13.083508000 | -0.114893000 | 1.763476000 |

| | | | |
|---|---|---|---|
| 6 | -6.197330000 | -0.819530000 | -1.375588000 |
| 1 | -6.558642000 | -1.858400000 | -1.379370000 |
| 1 | -5.519457000 | -0.730937000 | -2.231129000 |
| 6 | -5.485155000 | -4.032745000 | 0.004600000 |
| 7 | -5.448903000 | -2.878293000 | 0.648506000 |
| 7 | -6.412115000 | -2.823805000 | 1.609691000 |
| 6 | -4.544413000 | -1.705879000 | 0.417246000 |
| 7 | -6.485383000 | -4.760456000 | 0.546740000 |
| 1 | -4.838327000 | -4.347376000 | -0.801223000 |
| 6 | -7.030656000 | -3.971619000 | 1.532494000 |
| 1 | -7.863111000 | -4.269011000 | 2.154695000 |
| 6 | -6.937148000 | -6.115995000 | 0.156013000 |
| 1 | -7.933762000 | -6.041609000 | -0.292102000 |
| 1 | -7.003145000 | -6.731961000 | 1.058528000 |
| 1 | -3.880833000 | -2.044955000 | -0.386447000 |
| 1 | -11.082932000 | -2.280041000 | 2.343127000 |
| 1 | -1.617194000 | -1.639884000 | 3.474792000 |
| 6 | -3.732965000 | -1.446568000 | 1.712186000 |
| 1 | -4.206875000 | -0.627738000 | 2.264311000 |
| 1 | -3.796265000 | -2.330312000 | 2.358963000 |
| 6 | 12.398315000 | 0.370830000 | 1.917798000 |
| 1 | 12.597301000 | -0.138490000 | 2.868728000 |
| 1 | 13.353765000 | 0.379642000 | 1.378683000 |
| 6 | 12.937924000 | 5.562194000 | -1.526595000 |
| 7 | 13.869372000 | 6.211845000 | -1.293849000 |
| 6 | -6.001494000 | -6.714229000 | -0.794646000 |
| 7 | -5.238314000 | -7.146464000 | -1.552553000 |

Oligomer 3

| | | | |
|---|---|---|---|
| 6 | 6.351904000 | 1.239974000 | 0.159202000 |
| 6 | 6.391295000 | 1.607790000 | -1.196815000 |
| 6 | 6.474671000 | 2.269014000 | 1.105856000 |
| 1 | 6.279213000 | 0.837483000 | -1.956144000 |
| 1 | 6.446072000 | 2.038180000 | 2.166471000 |
| 6 | 6.582248000 | 2.928104000 | -1.594332000 |
| 6 | 6.653094000 | 3.594662000 | 0.714794000 |
| 1 | 6.596445000 | 3.167553000 | -2.654111000 |
| 1 | 6.744277000 | 4.367504000 | 1.475248000 |
| 6 | 6.739110000 | 3.952587000 | -0.642419000 |
| 6 | 7.019766000 | 5.354151000 | -1.002405000 |
| 1 | 6.867853000 | 6.073535000 | -0.197154000 |
| 6 | 7.456796000 | 5.812075000 | -2.186083000 |
| 1 | 7.633431000 | 5.160481000 | -3.038655000 |
| 1 | 7.619805000 | 6.872661000 | -2.351043000 |
| 6 | 6.220214000 | -0.227813000 | 0.551519000 |
| 1 | 6.335730000 | -0.800426000 | -0.377715000 |
| 6 | 4.794278000 | -0.627224000 | 1.043315000 |
| 1 | 4.123992000 | -0.497181000 | 0.184191000 |
| 1 | 4.813630000 | -1.707142000 | 1.246082000 |
| 6 | 8.840476000 | -1.696748000 | -0.373620000 |
| 6 | 8.784699000 | -3.076206000 | -0.111616000 |
| 6 | 8.953707000 | -1.297775000 | -1.710442000 |
| 1 | 8.696591000 | -3.429025000 | 0.913260000 |
| 1 | 8.984753000 | -0.236438000 | -1.949836000 |

| | | | |
|---|---|---|---|
| 6 | 8.851580000 | -4.010820000 | -1.136805000 |
| 6 | 9.018025000 | -2.233379000 | -2.743008000 |
| 1 | 8.808406000 | -5.067280000 | -0.889764000 |
| 1 | 9.104954000 | -1.891763000 | -3.772128000 |
| 6 | 8.973983000 | -3.611410000 | -2.481546000 |
| 6 | 9.053098000 | -4.559026000 | -3.604564000 |
| 1 | 9.090540000 | -4.092063000 | -4.588742000 |
| 6 | 9.089012000 | -5.895756000 | -3.539272000 |
| 1 | 9.065637000 | -6.443627000 | -2.601162000 |
| 1 | 9.149511000 | -6.495819000 | -4.441720000 |
| 6 | 8.761992000 | -0.666638000 | 0.744836000 |
| 1 | 8.860173000 | 0.323769000 | 0.280374000 |
| 6 | 7.386665000 | -0.685959000 | 1.463512000 |
| 1 | 7.176582000 | -1.699844000 | 1.827497000 |
| 1 | 7.453652000 | -0.048271000 | 2.351585000 |
| 6 | 2.651413000 | -0.275258000 | 2.319385000 |
| 6 | 2.234955000 | -1.578571000 | 2.625669000 |
| 6 | 1.658555000 | 0.691157000 | 2.111561000 |
| 1 | 2.972768000 | -2.358245000 | 2.798476000 |
| 1 | 1.948902000 | 1.712413000 | 1.874320000 |
| 6 | 0.881361000 | -1.895077000 | 2.737233000 |
| 6 | 0.304455000 | 0.376048000 | 2.223991000 |
| 1 | 0.591532000 | -2.911614000 | 2.996061000 |
| 1 | -0.439305000 | 1.155965000 | 2.072819000 |
| 6 | -0.108279000 | -0.922821000 | 2.547064000 |
| 6 | -1.578344000 | -1.259765000 | 2.692128000 |
| 1 | -2.082303000 | -0.462032000 | 3.254215000 |
| 6 | -2.305454000 | -1.440980000 | 1.344692000 |
| 1 | -1.823762000 | -2.249904000 | 0.776907000 |
| 1 | -2.194071000 | -0.530361000 | 0.748677000 |
| 6 | 4.130782000 | 0.096293000 | 2.243479000 |
| 1 | 4.172020000 | 1.175087000 | 2.047322000 |
| 6 | 4.812572000 | -0.163154000 | 3.602212000 |
| 1 | 4.885174000 | -1.235725000 | 3.816788000 |
| 1 | 5.825736000 | 0.248149000 | 3.639419000 |
| 6 | 12.038134000 | -1.832911000 | 0.721442000 |
| 6 | 12.315117000 | -2.850463000 | 1.649401000 |
| 6 | 12.484107000 | -2.010764000 | -0.591508000 |
| 1 | 11.986373000 | -2.750320000 | 2.681949000 |
| 1 | 12.273400000 | -1.249827000 | -1.339572000 |
| 6 | 13.003643000 | -3.998874000 | 1.277861000 |
| 6 | 13.171411000 | -3.163122000 | -0.967518000 |
| 1 | 13.195392000 | -4.762759000 | 2.025276000 |
| 1 | 13.496021000 | -3.278831000 | -1.998707000 |
| 6 | 13.450424000 | -4.183177000 | -0.043932000 |
| 6 | 14.182644000 | -5.378112000 | -0.492973000 |
| 1 | 14.438399000 | -5.380366000 | -1.552161000 |
| 6 | 14.556171000 | -6.427785000 | 0.249173000 |
| 1 | 14.346426000 | -6.507252000 | 1.312488000 |
| 1 | 15.097822000 | -7.257599000 | -0.193516000 |
| 6 | 11.330504000 | -0.549084000 | 1.144733000 |
| 1 | 11.194884000 | 0.052185000 | 0.234299000 |
| 6 | 9.922443000 | -0.777083000 | 1.766529000 |
| 1 | 9.729736000 | -0.032250000 | 2.549507000 |
| 1 | 9.895142000 | -1.749976000 | 2.270409000 |
| 6 | 12.318784000 | 3.175821000 | 0.523047000 |
| 7 | 11.515946000 | 2.538542000 | 1.365887000 |
| 7 | 10.209908000 | 2.754357000 | 1.048755000 |
| 6 | 11.859505000 | 1.650793000 | 2.501604000 |
| 7 | 11.539431000 | 3.822048000 | -0.363589000 |
| 1 | 13.398839000 | 3.175940000 | 0.540124000 |
| 6 | 10.244376000 | 3.534676000 | -0.001492000 |
| 1 | 9.368976000 | 3.909556000 | -0.515762000 |
| 6 | 11.970871000 | 4.697911000 | -1.474835000 |
| 1 | 13.055898000 | 4.603489000 | -1.562776000 |
| 1 | 11.519978000 | 4.322470000 | -2.396327000 |
| 1 | 12.654680000 | 2.145751000 | 3.066198000 |
| 1 | 10.959169000 | 1.642879000 | 3.116086000 |
| 1 | 4.237652000 | 0.297635000 | 4.412310000 |
| 6 | -9.063037000 | 1.979766000 | 0.526788000 |
| 6 | -9.039672000 | 3.351107000 | 0.224431000 |

| | | | | |
|---|---|---|---|---|
| 6 | -8.590854000 | 1.595523000 | 1.791393000 | |
| 1 | -9.405578000 | 3.685651000 | -0.743858000 | |
| 1 | -8.597172000 | 0.547648000 | 2.080933000 | |
| 6 | -8.566491000 | 4.294080000 | 1.130802000 | |
| 6 | -8.120213000 | 2.538302000 | 2.704971000 | |
| 1 | -8.576097000 | 5.343751000 | 0.852472000 | |
| 1 | -7.775356000 | 2.208844000 | 3.683091000 | |
| 6 | -8.088347000 | 3.908627000 | 2.395617000 | |
| 6 | -7.568786000 | 4.858764000 | 3.392365000 | |
| 1 | -7.328118000 | 4.414940000 | 4.358614000 | |
| 6 | -7.365530000 | 6.172737000 | 3.234079000 | |
| 1 | -7.574059000 | 6.695372000 | 2.304432000 | |
| 1 | -6.982277000 | 6.779449000 | 4.048515000 | |
| 6 | -9.604665000 | 0.982285000 | -0.491367000 | |
| 1 | -9.684451000 | 1.525992000 | -1.442274000 | |
| 6 | -11.073253000 | 0.545665000 | -0.200042000 | |
| 1 | -11.683170000 | 1.454427000 | -0.265915000 | |
| 1 | -11.398499000 | -0.093546000 | -1.033192000 | |
| 6 | -7.741883000 | 0.475147000 | -3.065892000 | |
| 6 | -8.268468000 | -0.547308000 | -3.873837000 | |
| 6 | -7.527890000 | 1.723813000 | -3.660304000 | |
| 1 | -8.454311000 | -1.532140000 | -3.449348000 | |
| 1 | -7.120105000 | 2.537034000 | -3.064148000 | |
| 6 | -8.568043000 | -0.331352000 | -5.213608000 | |
| 6 | -7.829956000 | 1.944155000 | -5.003467000 | |
| 1 | -8.975617000 | -1.147861000 | -5.802527000 | |
| 1 | -7.657284000 | 2.927195000 | -5.435128000 | |
| 6 | -8.357006000 | 0.924959000 | -5.812268000 | |
| 6 | -8.661315000 | 1.212111000 | -7.223249000 | |
| 1 | -8.427740000 | 2.228979000 | -7.537641000 | |
| 6 | -9.183684000 | 0.376328000 | -8.129190000 | |
| 1 | -9.451965000 | -0.652007000 | -7.901824000 | |
| 1 | -9.367926000 | 0.703497000 | -9.147515000 | |
| 6 | -7.390182000 | 0.239999000 | -1.603395000 | |
| 1 | -7.018188000 | 1.191197000 | -1.203376000 | |
| 6 | -8.621180000 | -0.180614000 | -0.759364000 | |
| 1 | -9.150286000 | -0.987529000 | -1.283221000 | |
| 1 | -8.276910000 | -0.608986000 | 0.188622000 | |
| 6 | -12.953188000 | -0.136067000 | 1.326527000 | |
| 6 | -13.807989000 | -0.854066000 | 0.473301000 | |
| 6 | -13.539460000 | 0.599827000 | 2.361618000 | |
| 1 | -13.392102000 | -1.436475000 | -0.345983000 | |
| 1 | -12.906891000 | 1.169781000 | 3.038914000 | |
| 6 | -15.186645000 | -0.837308000 | 0.648437000 | |
| 6 | -14.921825000 | 0.617958000 | 2.540209000 | |
| 1 | -15.812288000 | -1.404831000 | -0.034332000 | |
| 1 | -15.347956000 | 1.199525000 | 3.354461000 | |
| 6 | -15.778510000 | -0.098911000 | 1.689996000 | |
| 6 | -17.230661000 | -0.045742000 | 1.922775000 | |
| 1 | -17.529416000 | 0.571567000 | 2.769844000 | |
| 6 | -18.191547000 | -0.659178000 | 1.220799000 | |
| 1 | -17.985015000 | -1.292062000 | 0.361802000 | |
| 1 | -19.237024000 | -0.543630000 | 1.488754000 | |
| 6 | -11.439722000 | -0.145880000 | 1.138420000 | |
| 1 | -11.018018000 | 0.456102000 | 1.952363000 | |
| 6 | -10.890157000 | -1.580415000 | 1.283216000 | |
| 1 | -11.217320000 | -2.217289000 | 0.451978000 | |
| 1 | -9.794901000 | -1.598441000 | 1.308664000 | |
| 6 | -4.549911000 | 0.616935000 | -0.192398000 | |
| 6 | -3.569041000 | 0.788700000 | -1.184800000 | |
| 6 | -4.707543000 | 1.634133000 | 0.755446000 | |
| 1 | -3.423882000 | 0.028448000 | -1.949370000 | |
| 1 | -5.480023000 | 1.550591000 | 1.515555000 | |
| 6 | -2.769836000 | 1.924847000 | -1.218059000 | |
| 6 | -3.910086000 | 2.777370000 | 0.718684000 | |
| 1 | -2.025297000 | 2.021358000 | -2.002319000 | |
| 1 | -4.067782000 | 3.557581000 | 1.459043000 | |
| 6 | -2.921056000 | 2.948776000 | -0.262876000 | |
| 6 | -2.109075000 | 4.176308000 | -0.256109000 | |
| 1 | -2.363854000 | 4.882125000 | 0.533776000 | |
| 6 | -1.122245000 | 4.500902000 | -1.100536000 | |

| | | | | |
|---|---|---|---|---|
| 1 | -0.805562000 | 3.858395000 | -1.917776000 | |
| 1 | -0.593609000 | 5.443668000 | -1.001366000 | |
| 6 | -5.437781000 | -0.621059000 | -0.148395000 | |
| 1 | -6.145430000 | -0.484141000 | 0.677683000 | |
| 6 | -6.240973000 | -0.795886000 | -1.461534000 | |
| 1 | -6.677488000 | -1.803227000 | -1.521570000 | |
| 1 | -5.559518000 | -0.706452000 | -2.314788000 | |
| 6 | -5.616961000 | -4.134132000 | -0.398792000 | |
| 7 | -5.541295000 | -3.045544000 | 0.352801000 | |
| 7 | -6.505665000 | -3.051977000 | 1.312373000 | |
| 6 | -4.605302000 | -1.885017000 | 0.231773000 | |
| 7 | -6.643829000 | -4.876264000 | 0.061053000 | |
| 1 | -4.982053000 | -4.377404000 | -1.237560000 | |
| 6 | -7.159003000 | -4.168935000 | 1.119437000 | |
| 1 | -8.005847000 | -4.497823000 | 1.704830000 | |
| 6 | -7.082031000 | -6.203686000 | -0.417800000 | |
| 1 | -6.868040000 | -6.258655000 | -1.488238000 | |
| 1 | -8.164766000 | -6.260411000 | -0.287540000 | |
| 1 | -3.945191000 | -2.165869000 | -0.597113000 | |
| 1 | -11.256063000 | -2.034045000 | 2.210861000 | |
| 1 | -1.687054000 | -2.175046000 | 3.288882000 | |
| 1 | -6.578530000 | -7.222929000 | 1.428619000 | |
| 1 | -5.325981000 | -7.356069000 | 0.183652000 | |
| 6 | -6.412187000 | -7.361116000 | 0.354488000 | |
| 1 | 12.025528000 | 6.572156000 | -0.355393000 | |
| 1 | 10.465495000 | 6.220303000 | -1.124898000 | |
| 6 | 11.551614000 | 6.168369000 | -1.260801000 | |
| 6 | -3.794538000 | -1.766569000 | 1.547708000 | |
| 1 | -4.261835000 | -1.002648000 | 2.178825000 | |
| 1 | -3.872814000 | -2.710357000 | 2.101301000 | |
| 6 | 12.293961000 | 0.242051000 | 2.070069000 | |
| 1 | 12.463853000 | -0.312855000 | 3.001089000 | |
| 1 | 13.273296000 | 0.307132000 | 1.579137000 | |
| 8 | 11.857162000 | 6.902953000 | -2.420984000 | |
| 1 | 12.753928000 | 7.264768000 | -2.352925000 | |
| 8 | -7.022394000 | -8.573612000 | -0.008656000 | |
| 1 | -6.569429000 | -8.953956000 | -0.776669000 | |
